# *Bordetella bronchiseptica* diguanylate cyclase BdcB inhibits the type three secretion system and impacts the immune response

**DOI:** 10.1038/s41598-023-34106-x

**Published:** 2023-05-02

**Authors:** Keila Belhart, Federico Sisti, Mónica C. Gestal, Julieta Fernández

**Affiliations:** 1 Departamento de Ciencias Biológicas, Facultad de Ciencias Exactas, Instituto de Biotecnología y Biología Molecular (IBBM)-CCT-CONICET-La Plata, Universidad Nacional de La Plata, La Plata, Argentina; 2 Department of Microbiology and Immunology, Louisiana State University (LSU) Health Sciences Center at Shreveport, Shreveport, LA USA

**Keywords:** Immunology, Microbiology, Molecular biology, Pathogenesis

## Abstract

*Bordetella bronchiseptica* is a gram-negative bacterium that causes respiratory diseases in different animals, including mice, making *B. bronchiseptica* the gold-standard model to investigate host–pathogen interaction at the molecular level. *B. bronchiseptica* utilizes many different mechanisms to precisely regulate the expression of virulence factors. Cyclic di-GMP is a second messenger synthesized by diguanylate cyclases and degraded by phosphodiesterases that regulates the expression of multiple virulence factors including biofilm formation. As in other bacteria, we have previously shown that c-di-GMP regulates motility and biofilm formation in *B*. *bronchiseptica*. This work describes the diguanylate cyclase BdcB (*Bordetella* diguanylate cyclase B) as an active diguanylate cyclase that promotes biofilm formation and inhibits motility in *B*. *bronchiseptica*. The absence of BdcB increased macrophage cytotoxicity in vitro and induced a greater production of TNF-α, IL-6, and IL-10 by macrophages. Our study reveals that BdcB regulates the expression of components of T3SS, an important virulence factor of *B. bronchiseptica*. The *Bb*∆*bdcB* mutant presented increased expression of T3SS-mediated toxins such as *bteA*, responsible for cytotoxicity. Our in vivo results revealed that albeit the absence of *bdcB* did not affect the ability of *B. bronchiseptica* to infect and colonize the respiratory tract of mice, mice infected with *Bb*∆*bdcB* presented a significantly﻿ higher pro-inflammatory response than those infected with wild type *B. bronchiseptica*.

## Introduction

*Bordetella bronchiseptica* is a Gram-negative respiratory pathogen frequently found in pets like dogs or cats, in farm animals like pigs, and is increasingly found in humans, especially immunocompromised or with cystic fibrosis^1,2^. The expression of virulence factors and biofilm formation is regulated by a two-component system, BvgAS^3–5^. The histidine kinase, BvgS, has been postulated to be constitutively active but inhibited by low temperatures, magnesium sulfate, or nicotinic acid^6,7^. If *B. bronchiseptica* is grown in presence of these compounds or at temperatures below 25 °C, biofilm formation is impaired, and the expression of virulence factors is suppressed^5,8^. However, during these conditions, the flagellar apparatus is expressed, and the bacteria exhibit motility^9^. We have previously described that the second messenger bis-(3′-5′)-cyclic dimeric guanosine monophosphate (c-di-GMP) regulates biofilm and motility in *B. bronchiseptica*^10,11^. C-di-GMP is extensively associated with the transition between planktonic and biofilm lifestyles in bacteria; when c-di-GMP levels are high a biofilm-forming phenotype is present in most bacteria^12^. On the contrary, when c-di-GMP levels drop, a planktonic lifestyle is predominant, and bacteria usually exhibit flagella-mediated motility. Also, in pathogenic bacteria, some virulence factors are regulated by c-di-GMP levels, including the type three secretion system (T3SS) which is repressed by high levels of c-di-GMP. High levels of the second messenger achieved by deletion of phosphodiesterases in *Dickeya dadantii* inhibit T3SS expression and, in consequence, the virulence of the pathogen^13^. Similarly, *Pseudomonas aeruginosa* T3SS is inhibited by c-di-GMP^14^.

C-di-GMP concentration in the bacterial cytosol is regulated by two types of enzymes: diguanylate cyclases (DGCs) that synthesize c-di-GMP from two molecules of GTP and phosphodiesterases (PDEs) that degrade c-di-GMP. One GTP molecule binds to a GGDEF domain present in the DGCs. Dimerization of two GGDEF domains bound to GTP initiates c-di-GMP synthesis. The process is facilitated by the N-term domain usually present in DGCs (reviewed in^15^). The diversity of domains present in DGCs may explain the variety of signals that can modulate c-di-GMP levels in bacteria (recently reviewed in^16^). Also, a different number of DGCs and PDEs are present in the bacteria genome, probably in response to different signals that regulate c-di-GMP concentration. Hence, not all the DGCs are active and responsible for all phenotypes regulated by c-di-GMP. Fluctuations in local concentration c-di-GMP also may explain phenotypes regulated only by one or two from dozen of DGCs present in a genome.

In *B. bronchiseptica* we described a particular DGC, BdcA, responsible for motility regulation and persistence in the host airway^11^. Constitutive overexpression of *bdcA* from a plasmid significantly increases c-di-GMP levels in *B. bronchiseptica*, affecting the expression of different genes that overall had an impact on the amount of functional protein^11,17^. Interestingly, among the genes regulated by c-di-GMP, we found the locus encoding the type three secretion system (T3SS) in *B. bronchiseptica*^18^. The T3SS is the main responsible for *B. bronchiseptica* cytotoxicity on immune cells^18–20^. Also, it is well established that *Bordetella* spp. utilizes T3SS amongst other virulence mechanisms to suppress host immune responses. The T3SS inhibits the generation of IFN-γ-producing splenocytes and stimulates the production of the immunosuppressive IL-10^21,22^.

In the present work, we show that a newly described DGC -BdcB- regulates the T3SS in *B. bronchiseptica*. DGC activity of BdcB is required for the synthesis of c-di-GMP which promotes T3SS inhibition. The immune response of mice infected with a *bdcB* mutant was the expected for an overexpressing T3SS bacteria, presenting increased IL-6, IL-10, and increased macrophage death. Overall, we described a DGC that contributes to the complex regulatory network of the T3SS function.

## Results

### BdcB is a putative diguanylate cyclase with a disordered N-term region

**Figure 1:**
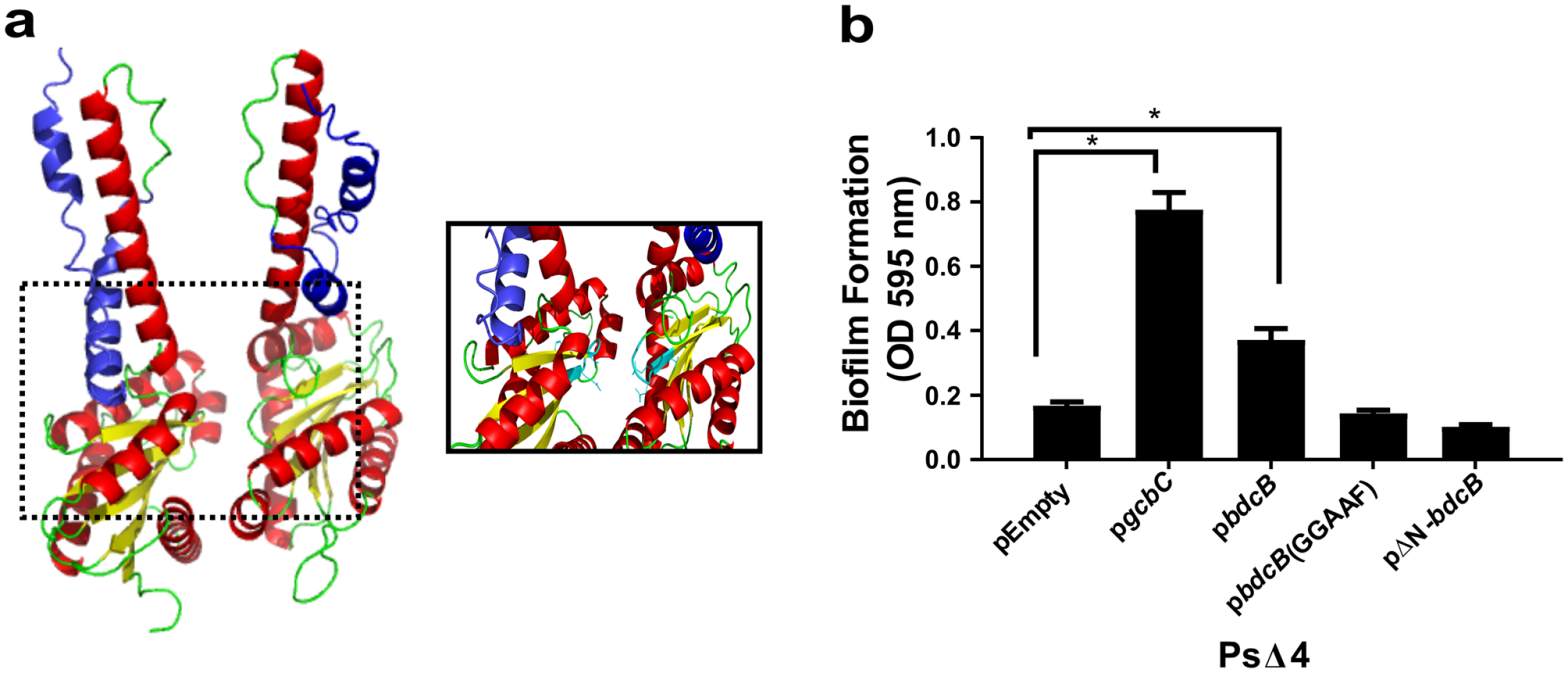
BB3903 (BdcB) is an active diguanilate cyclase. (**A**) ColabFold structure prediction of BB3903 dimer. Alpha helixs in red and β sheets in yellow. In blue, the predicted N term disordered region. GGDEF aminoacids from active site colored in cyan. (**B**) *P. fluorescens*Δ4 biofilm formation after 6 h incubation at 28 °C in static conditions and stained with crystal violet (CV) solution. pEmpty indicates original plasmid, p*gcbC* or p*bdcB* and variants indicate original plasmid expressing the indicated gene. Quantification was conducted by dissolving CV in acetic acid solution and measuring OD at 595 nm. The results are an average of three biologically independent replicates. ANOVA with a Tukey’s multiple-comparison test, * p < 0.05.

In *Bordetella bronchiseptica*, like other organisms, c-di-GMP is important during the infectious process^17^. *B. bronchiseptica* genome contains twelve putative DGCs. We previously described the active DGC BdcA, which regulates motility and is required to effectively persist in the mice respiratory tract^11^. To further characterize the c-di-GMP network in *B. bronchiseptica*, we decided to study the role of a cytosolic DGC, BB3903. This putative DGC is 251 amino acids long and has a predicted molecular weight of 27.6 kDa. Utilizing bioinformatics approaches provided in the Uniprot database, we found that BB3903 is devoided of regulatory domain, such as a phosphorylation receiver or sensing domain that are often associated with GGDEF domain. Stand-alone GGDEF proteins are rare in the genomes since DGCs need to dimerize to synthesize c-di-GMP, and the companion domain stabilizes the dimerization. We used a AlphaFold2_mmseqs2 based software located at ColabFold platform to model the probable BB3903 dimer structure (Fig. 1A and Fig. S1)^23^. As expected, the GGDEF domain presented the typical structure composed of a central five-stranded β sheet surrounded by five α helices and one hairpin. Also, important amino acids previously described for DGC activity are present^24^. The GGDEF domain is linked to the rest of the protein by a coiled-coil structure also present in other DGCs. Interestingly, a not-structured region, namely IDR (for the *i*ntrinsically disordered *r*egion) is predicted to be located between amino acids 1 and 46^25^. To date is the first time an IDR region has been described in a GGDEF-containing protein. IDR has been described as involved in the recognition of ligands and in protein–protein interactions^26^.

### BdcB restored biofilm defective *Pseudomonas fluorescens* mutant

Although the GGDEF domain has all the necessary amino acids for DGC activity, we proceeded to perform functional assays to evaluate the DGC activity in vitro. We cloned the *bb3903* gene in a replicative plasmid and express it in a biofilm defective *Pseudomonas fluorescens* strain (*Pf*Δ4). This heterologous expression system has been previously validated to evaluate DGC activities^10^. The main concept is that *Pf*Δ4 has four DGC deleted and it is not able to form biofilm on top of plastic surfaces unless an active DGC is overexpressed; allowing us to demonstrate that a particular protein functions as DGC. As shown in Fig. 1B, our positive control, GcbC, a well-known DGC from *P. fluorescens* can complement the defective phenotype present in *Pf*Δ4. BB3903 induced a partial restoration of the phenotype, evidenced by *Pf*Δ4-p*bdcB* biofilm formation visualized as a violet ring in the air–liquid interphase. An amino acid substitution in the active site (GGDEF to GGAAF) prevents complementation, indicating that BB3903 activity is required to promote biofilm formation on the *Pf*Δ4 strain. Moreover, our results show that the N-term IDR is important for BB3903 function, given that a truncated version of BB3903 was not able to revert the defective biofilm phenotype of *Pf*Δ4 (Fig. 1B, far-right column).

In conclusion, we demonstrated that BB3903, hereafter BdcB(*Bordetella* diguanylate cyclase B), is an active DGC and the N-term portion, predicted to be an IDR, is required for activity.

### Overexpression of *bdcB* inhibits motility and enhances biofilm formation in *Bordetella bronchiseptica*

**Figure 2:**
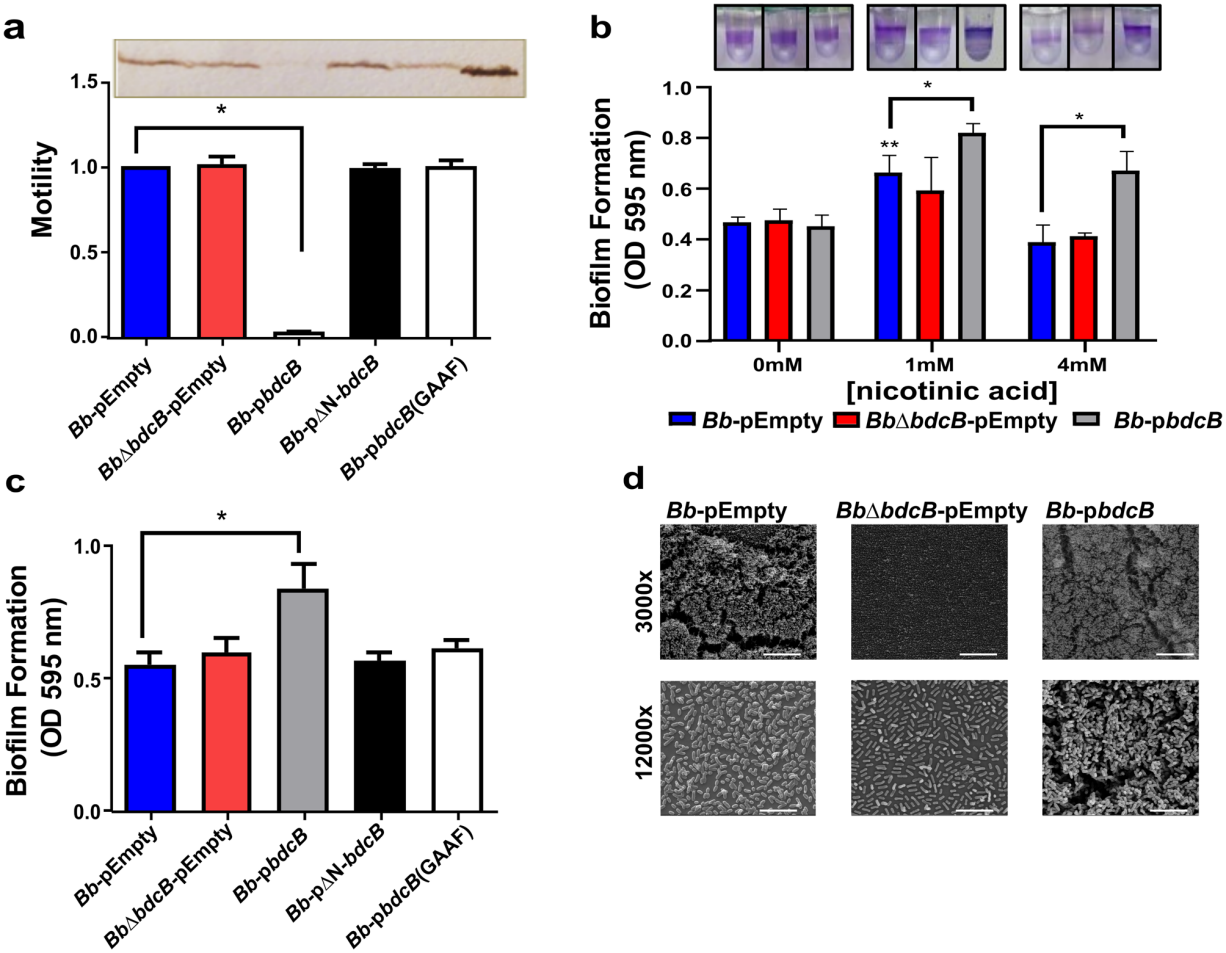
BdcB stimulates biofilm and inhibits motility in *B. bronchiseptica*. Swimming motility and flagella expression (**A**), biofilm formation on PVC U bottom wells with different nicotinic acid concentrations (**B**), biofilm formation on PVC U bottom wells with 1 mM nicotinic acid (**C**), and biofilm formation over glass observed by SEM (**D**). of *B. bronchiseptica* strains. Motility assay with soft agar (0.35%) was used to quantify the motility of *B. bronchiseptica* strains, motility is relative Bb-pEmpty. Western blot with polyclonal sera against flagellin was employed to determine flagella presence in whole bacteria. Purified flagellin (far right line) was incorporated as a positive control. pEmpty indicates original plasmid, p*bdcB* and variants indicate original plasmid expressing the indicated gene. A representative result from two independent experiments is shown. *B. bronchiseptica* biofilm formation after 24 h incubation at 37 °C in static conditions and stained with crystal violet (CV) solution. Quantification was conducted by dissolving CV in acetic acid solution and measuring at 595 nm. The results are an average of five biologically independent experiments, with 6 technical replicates each. ANOVA with a Tukey’s multiple-comparison test, *,* p *< 0.05 vs pEmpty at the same nicotinic acid concentration; ** vs pEmpty at 0 mM nicotinic acid. SEM was performed after 24 h of incubation at 37 °C with SS + 1 mM nicotinic acid. Coverslips were then treated to perform a CO_2_-critical-point procedure (EmiTech K850) and sputter coated with gold. Most of the sample was scanned and representative images were selected for processing. White bars indicate 20 µm (3000×) or 5 µm (12000×).

Phenotypes like swimming motility and biofilm formation are regulated by c-di-GMP in *B. bronchiseptica*^10^. Motility in *B. bronchiseptica* can be evaluated in soft agar SS plates supplemented with MgSO_4_. This media derepresses the flagellar system and, therefore, the bacteria are able to swim. To evaluate the BdcB role in these phenotypes we deleted the *bdcB* gene by double recombination as previously described^27^. No differences in motility or flagella production were observed in the mutant strain as compared to the parental strain in the condition tested (Fig. 2A and Fig. S2). However, overexpression of *bdcB* significantly inhibits motility and the protein flagellin was downregulated in the *Bb*-p*bdcB* strain, as expected for a DGC function. If an active site mutant (GGDEF to GGAAF) or the delta N-term version were overexpressed, *B. bronchiseptica* presented a motility phenotype indistinguishable from wild type *Bb*. We confirmed that both mutated and truncated versions were expressed, detecting HA-tagged proteins on a western blot (Fig. S3).

*B. bronchiseptica* forms biofilm over plastic surfaces. This phenotype is regulated by the two-component system BvgAS^20^. When the system is partially active, bacteria are in an intermediate virulence phase and biofilm formation is maximal. As expected, the wild type strain presented this behavior (Fig. 2B). Consistent with *P. fluorescens* experiments, *bdcB* overexpression increased biofilm formation in intermediate and avirulent phases (with 1 or 4 mM nicotinic acid respectively) (Fig. 2B) but overexpression of mutant versions of *bdcB* (*bdcB*-GGAAF or ΔN-*bdcB*) did not modify biofilm formation (Fig. 2C). Although *bdcB* is present in all phases at the same level (Fig. S4), biofilm formation by the *bdcB* mutant (*Bb*Δ*bdcB*) revealed no significant differences from the parental strain in any virulent phase evaluated (Fig. 2B).However, differences in biofilm formation on glass surface were evident when they were observed by scanning electronic microscopy (SEM) (Fig. 2D). Differences in biofilm were not due to differences in growth (Fig. S5).

Thus, our results indicate that BdcB is an active DGC that inhibits flagella expression and motility. In the wild type strain, BdcB is involved in biofilm regulation, probably in the first steps of the process.

### BdcB is not required for colonization of the mice’s respiratory tract

Our previous results demonstrate that BdcB mediates biofilm formation. We have previously proven that high c-di-GMP levels are detrimental to *B. bronchiseptica* infection in the mouse model^17^. Besides, it has been proposed that biofilm formation is critical for the first steps of the infection^28,29^. Considering that the *bdcB* mutation induced a mild defect in biofilm formation, we hypothesized that the adhesion and colonization process in the upper respiratory tract would be defective in the *bdcB*-null mutant. To evaluate this, we infected groups of BALB/c and C57BL/6 J mice with the knockout mutant strain. As a control, other groups were inoculated with the wild type *Bb* or with a mutant with the complete *bdcB* gene reinserted in the original location in the genome (reconstituted strain, *Bb*Δ*bdcB*^*r*^). Inoculations were performed using the classical high dose high volume challenge^30^. We used both animal models, for rigor and reproducibility purposes, as it has been shown that there are subtle differences in the immune response of both mouse models, that can impact our results^31^.

**Figure 3:**
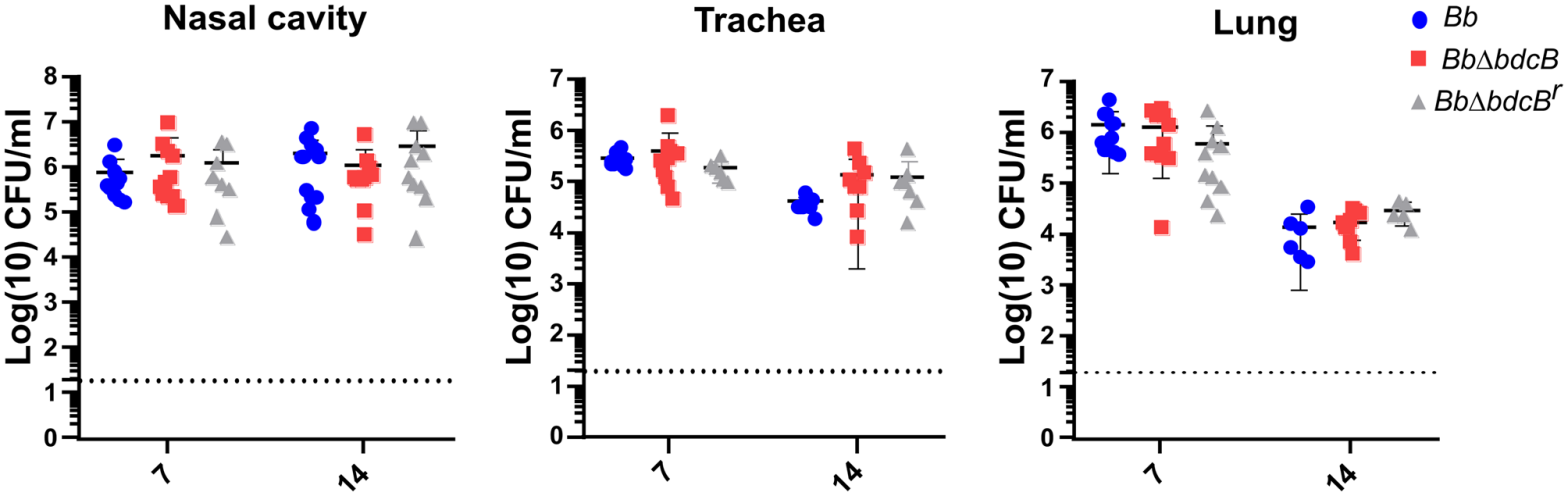
BdcB is not required for mice colonization. BALB/c mice were intranasally inoculated with 30 μL of 1 × 10^6^ CFU/mL of *Bb* (blue circles), *Bb*Δ*bdcB* (red squares) and *Bb*Δ*bdcB*^r^ (gray triangles) 7 and 14 days post-infection, mice were euthanized, nasal cavity, trachea, and lungs were homogenized, and the number of viable bacteria was determined by serial dilution counts. Each symbol represents a single animal, with the mean colonization depicted as short horizontal bars. Data were pooled from 2 separate experiments conducted independently with 5 animals per day in each experiment. A dashed line indicates the limit of detection. ANOVA with a Tukey’s multiple-comparison test, no significative differences were observed.

Accordingly to previously published literature, wild type *B. bronchiseptica* colonized mice respiratory track and persisted for 14 days post-infection (Fig. 3). The *bdcB* mutants, both knocked out and complemented, behaved similarly to the wild type; no differences were observed in the number of bacteria recovered from the different organs at different times post-infection (Fig. 3). The results were reproducible using both murine models C57BL/6 J and BALB/c (Fig. 3 and Fig. S6), indicating the subtle differences in immune responses between these two animal strains did not impact clearance of the infection caused by any of these three strains. Thus, *bdcB* has no impact on colonization and infection of the respiratory tract.

### Mice immune response is dependent on BdcB presence

**Figure 4:**
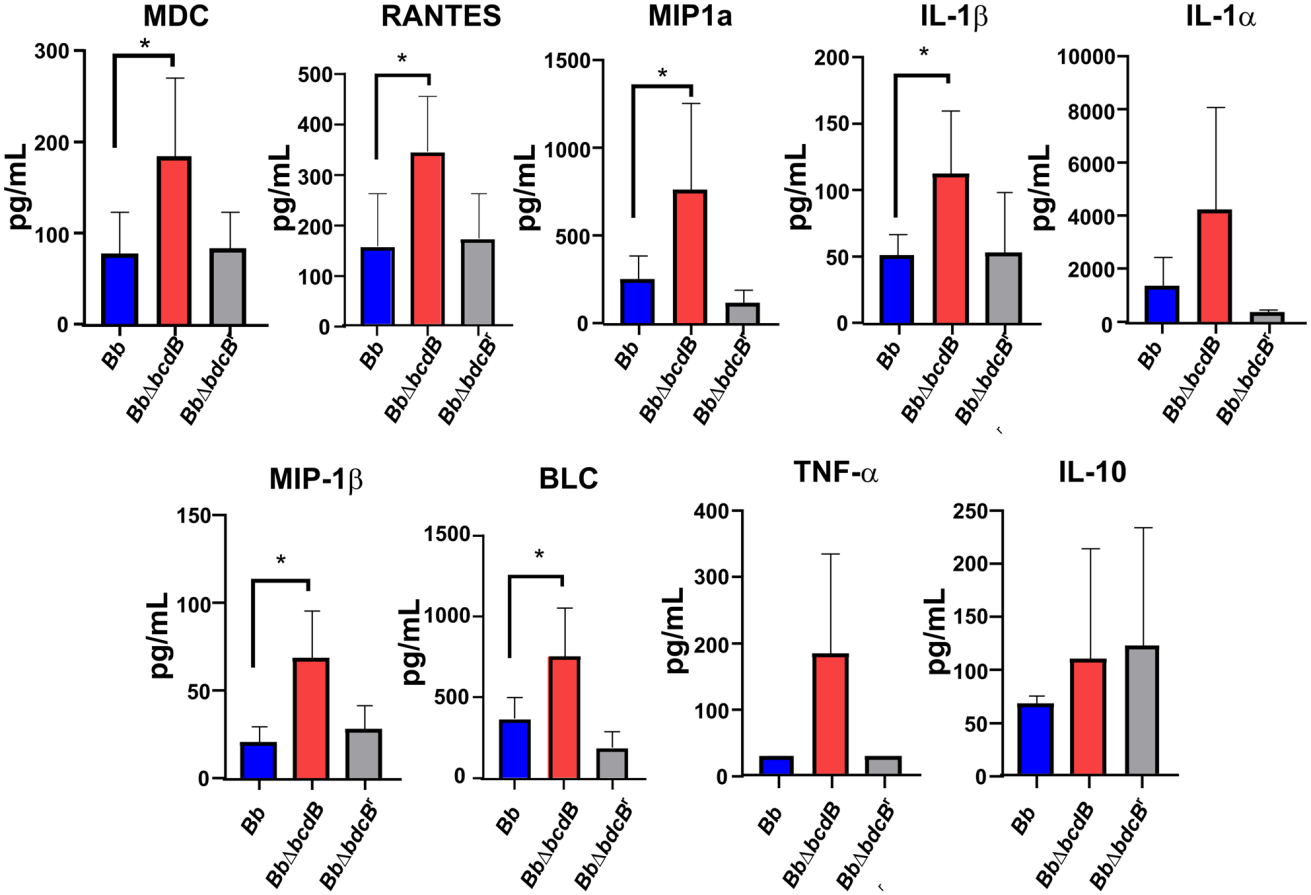
BdcB regulates immune response during infection. Murine cytokines were measured using LEGENDplex™ (BioLegend) bead-based immunoassays in homogenates lung supernatants 7 days post-infection. Group comparisons were analyzed by one-way ANOVA followed by Tukey’s multiple-comparison test. *,* p *< 0.05

Our previous results indicate that *bdcB* does not affect colonization and clearance of the infection. However, we previously showed that high levels of c-di-GMP have a significant impact on immune response, *B. bronchiseptica* overexpressing a DGC induce lower levels of proinflammatory cytokines (IL-6, IL-5, IL-1β, and TNF-α) and higher amounts of IL-10 in the infected mice^17^. We then decided to investigate the cytokine secretome at day 7 post-infection, coinciding with the peak of infection since we observed the maximal difference in immune response in a previous work^17^. We used lung homogenates of BALB/c and C57BL/6 J mice infected with the three bacterial strains. Significant differences in the cytokine profiling of mice were detected in the lungs (Fig. 4 and Fig. S7). We observed a stronger inflammatory response triggered by the *bdcB*-null mutant compared to that elicited after infection of either BALB/c or C57BL/6 mice with *Bb* or complemented strain. Cytokines such as IL-1β appeared secreted in higher amounts following infection with the knockout mutant strain, suggesting the possibility of a stronger inflammatory response. Chemokines that promote cellular recruitment to the lungs such as RANTES also known as CCL5, MIP-1α also known as CCL3, MIP-1β or CCL4, MDC or CCL22, and CXCL13 also known as BLC, were significantly increased in the lungs of the *Bb*Δ*bdcB* infected mice. Looking at the levels of immune signals in the lungs of mice infected with the complemented mutant, our results indicate that the levels of those cytokines and chemokines were reduced resembling the results obtained in mice infected with the wild type strain.

In summary, the absence of BdcB did not play a role in the ability of the bacteria to persist in the respiratory tract of mice in the period of infection analyzed. However, a stronger immune response was induced by this bacterial mutant, characterized by significant cell recruitment signaling.

### BdcB is important for surviving the bactericidal activity of macrophages

**Figure 5:**
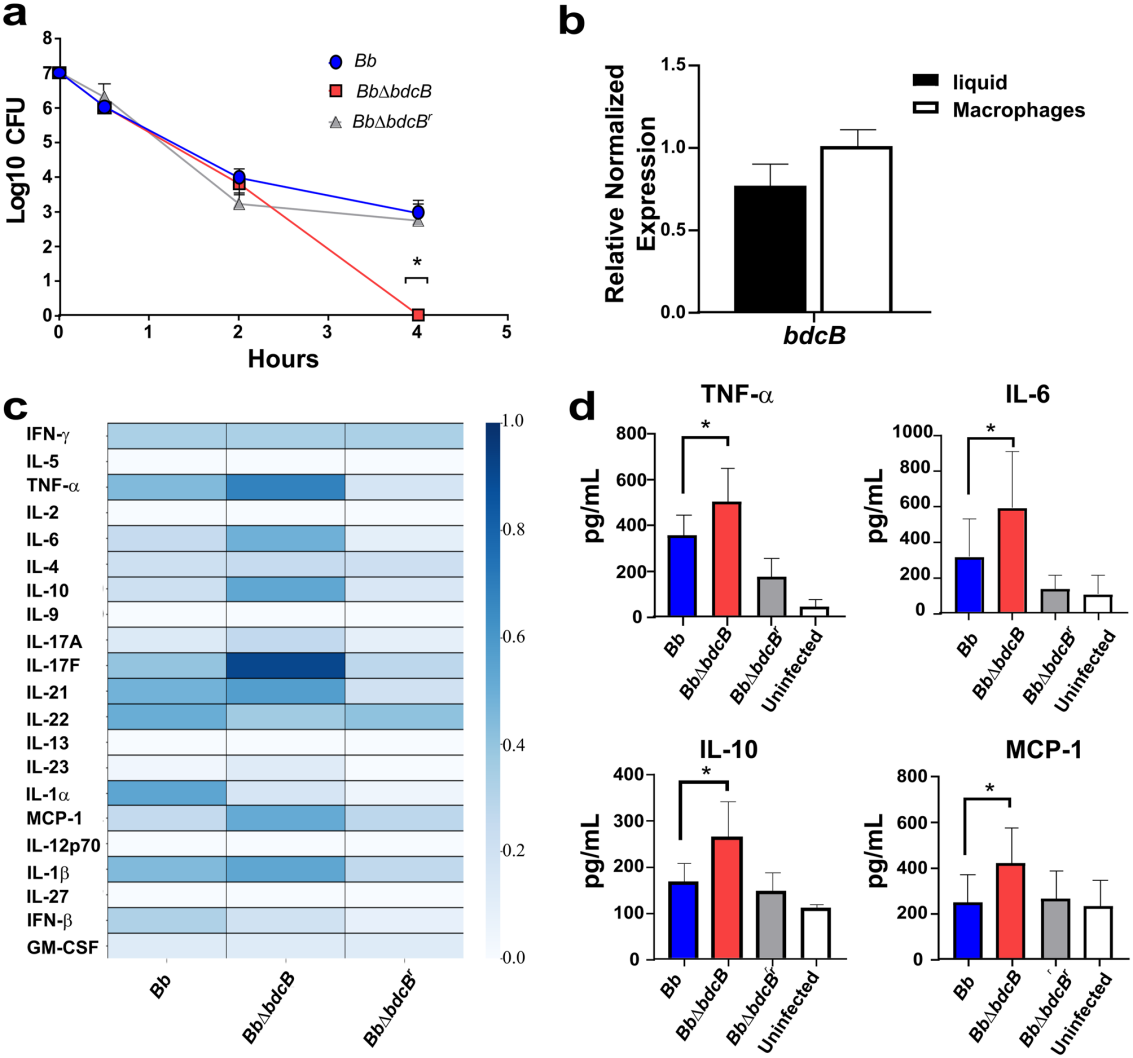
*Bb*Δ*bdcB* is eliminated by macrophages but induces a stronger inflammatory response. (**A**) Intracellular survival of *Bb*Δ*bdcB* in BALB/c derived macrophages. Bacteria and cells were co-cultivated in DMEM media (GIBCO) with MOI 100. At indicated points, viable intracellular bacteria were determined by serial dilutions of lysed macrophages. The average of three biological replicates is shown. Differences were analyzed by unpaired one-tailed Student's t-test. *,* p *< 0001. (**B**) *bdcB* mRNA amounts from free bacteria and bacteria inside the macrophages were determined by quantitative real-time PCR. Fold changes were calculated by the ΔΔCT method using *recA* levels as a control. Results are an average of three biologically independent experiments. No significant differences were observed. (**C**) Heat map panel of cytokines present in infected macrophages supernatants 4 h post-challenge. The concentration of each cytokine was determined by immunoassay. (**D**) Individual values for cytokines that presented significative differences are represented. Results are an average of two biologically independent experiments with six technical replicates. *,* p *< 0.05 (unpaired one-tailed Student's t-test).

The remarkable differences observed in the immune response to *B. bronchiseptica bdcB* mutant led us to analyze the interaction between bacteria and the immune response using a reductionist approach that allowed us to ask more mechanistic questions. We infected murine primary bone marrow-derived macrophages (BMM), obtained from BALB/c mice to determine the ability of the three bacteria to survive intracellularly (Fig. 5A). Consistent with previous reports, wild type *B. bronchiseptica* survives intracellularly for over 4 h post-infection^32^. Deletion of *bdcB* drastically impacted bacteria survival, and we could not recover viable bacteria at 4 h post-infection from BBM infected with the *bdcB* mutant. In our complemented strain where *bdcB* was restored, survival dynamics within macrophages were found to be equal to *Bb*.

Due to the differences, we hypothesized that *bdcB* might be upregulated when internalized in macrophages, allowing *B. bronchiseptica* to modulate gene expression to adapt to the intracellular niche and leading to the observed differences identified when *bdcB* was not present. We analyzed the levels of expression of *bdcB* in a liquid culture medium and within macrophages by qRT-PCR. Our results revealed that no significant differences were observed in the expression of *bdcB* when bacteria were internalized in the macrophages (Fig. 5B), suggesting that BdcB activity and not levels of expression of mRNA might be driving those differences.

Considering the previously observed differences in the secretome profiling of the lungs of infected mice, we decided to investigate the contribution of macrophages to the observed differences. We incubated bacteria with the BALB/c mice´s derived macrophages to evaluate the concentration of different secreted cytokines after the challenge with the three different strains. Similar to our results in vivo, the concentration of pro-inflammatory cytokines secreted was higher in BMM infected with *Bb*Δ*bdcB* (Fig. 5C and D). Inflammatory cytokines such as TNF-α, IL-1β, and IL-6 were significantly increased when *bdcB* was absent, and they were restored when BdcB was complemented, indicating an important role for this DGC in the regulation of the immune response. A regulatory cytokine, IL-10, was also induced in supernatants from BMM infected with the mutant.

Overall, our results indicate that BdcB contributes to the ability of bacteria to persist within macrophages and to suppress cytokine responses by macrophages.

### BdcB contributes to pH resistance

**Figure 6:**
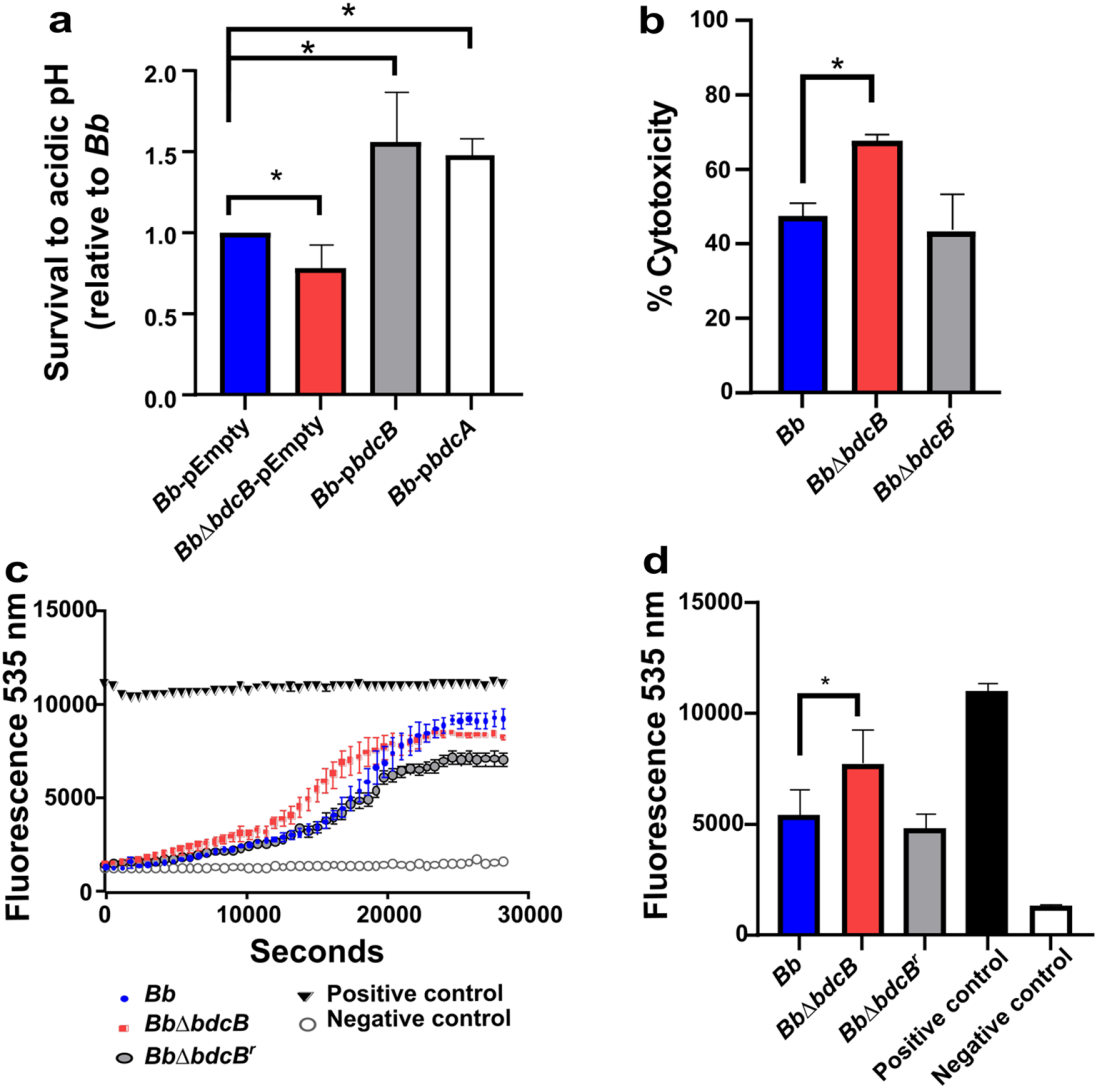
A high concentration of c-di-GMP is beneficial for acidic pH survival and inhibits toxic activity against macrophages. **(A**) Survival to acidic pH was determined as a ratio between viable bacteria present before and after being exposed to acidic pH for 4 h. pEmpty indicates the original plasmid, and p*bdcA* or p*bdcB* indicate original plasmid expressing the indicated gene. Results shown are means of three biologically independent experiments. *,* p *< 0.05 (unpaired one-tailed Student's t-test). (**B**) Cytotoxicity assays on bone-marrow-derived macrophages. *Bb*, *Bb*Δ*bdcB,* and *Bb*Δ*bdcB*^r^ strains were added at MOI = 1 on macrophage monolayers and then incubated in 5% CO_2_ at 37 °C. Cytotoxicity assays at 4 h were conducted using a Pierce LDH cytotoxicity assay kit, and results were expressed relative to the maximum LDH release control. The PI assay was performed in IP-containing media, fluorescence was measured at different time points at 535 nm (**C**), and statistical analysis was performed at 5 hs (18,000 s) (**D**) Three biologically independent experiments were performed. *,* p *< 0.05 (unpaired one-tailed Student’s t-test).

Once inside the macrophage, bacteria have to overcome different bactericidal strategies. Following phagocytosis, during the maturation to the phagosome, the pH of the vesicles becomes acid leading to bacterial death. Considering that *Bb*Δ*bdcB* was not recovered from BMMs at 4 h post-challenge, we wanted to evaluate resistance to acidic pH by incubating the three strains to pH 4 for 4 h and enumerating the viable bacteria. For the data analysis, the number of viable bacteria was determined and normalized to the initial number (Fig. 6A).

Our results revealed that more than half of *Bb* were killed by acidic pH (0.6 relative to *Bb*). Remarkably, the absence of *bdcB* was detrimental, and only 20% of mutants survived these conditions. Contrary, overexpression of *bdcB* enhances *B. bronchiseptica*’s resistance to acidic pH, confirming a role for BdcB in resistance to acidic stress. BdcB may induce stress resistance specifically, or because of increased levels of c-di-GMP. We overexpressed *bdcA* in the mutant to distinguish between these possibilities, and the acidic stress resistance phenotype was present, indicating that high levels of c-di-GMP induced by either BdcA or BdcB were sufficient to prevent death by low pH.

### BdcB inhibits macrophage death

After infection with *Bb*Δ*bdcB*, BMM supernatants presented higher cytokines levels, which is consistent with in vivo results. To assess whether this cytokine response is a consequence of eukaryotic cell death due to exacerbated bacterial toxicity, we measured levels of lactate dehydrogenase (LDH) enzyme release using a commercially available kit. We tested our three bacterial strains, Bb, *Bb*Δ*bdcB,* and ∆*bdcB*^r^ using BMMs from BALB/c or C57BL/6 mice to increase rigor and reproducibility. LDH measurement was performed on the supernatants of the infected cultures, 4 h post-infection using different multiplicities of infection (MOI): 1, 10, and 100. (Fig. 6B and Fig. S8).

Our first observation was that the percentage of cytotoxicity of *Bb* increases both in the infection of macrophages obtained from BALB/c mice and those obtained from C57BL/6 J mice proportionally to the multiplicity of infection used for the challenge. This was expected as it makes sense that more bacteria kill more macrophages. Importantly, at MOI:1, the cytotoxicity of *Bb*Δ*bdcB* was significantly greater than the cytotoxicity produced by *Bb*, indicating that the absence of *bdcB* could be associated with more cell death. However, at MOI:10 and MOI:100 no significant differences were observed between the tested strains, possibly due to a saturation effect, highlighting the importance of testing at different MOI to obtain more significant data and not overlook possible differences in phenotypes.

We performed a complementary assay in which we stained cells with propidium iodide (PI) and measured fluorescence in real-time during the infection experiment (Fig. 6C,D and Fig. S8). PI binds stoichiometrically to nucleic acids but is unable to cross cell membranes, so it only binds to the DNA of cells that have been killed. We incubated bacteria with the macrophages in PI-containing media and measured fluorescence as a function of time. These assays were performed using the three bacterial strains, Triton X100 as a positive control, and media as a negative control. For these in vitro experiments, we used macrophages obtained from BALB/c and C57BL/6 J mice.

During the first three hours of the assay, macrophages in contact with three strains tested showed a similar increase in fluorescence. Interestingly, after this point, the *Bb*Δ*bdcB* infected macrophages began to show increases in fluorescence that were greater than those observed for both *Bb* and *Bb*∆*bdcB*^r^ infected macrophages. After six hours all three curves reached a maximum value of fluorescence close to 10,000. This behavior was observed in both BALB/c (Fig. 6C and 6D) and C57BL/6 J (Fig. S8) derived macrophages. Thus, we conclude that the absence of *bdcB* promotes cytotoxicity and cytokine pro-inflammatory responses suggesting that although the effects of *bdcB* in colonization and infection might become indistinguishable in our assays, showing that *bdcB* plays a role in promoting cell death and pro-inflammatory responses in vitro and in vivo.

### BdcB inhibits the type three secretion system

**Figure 7:**
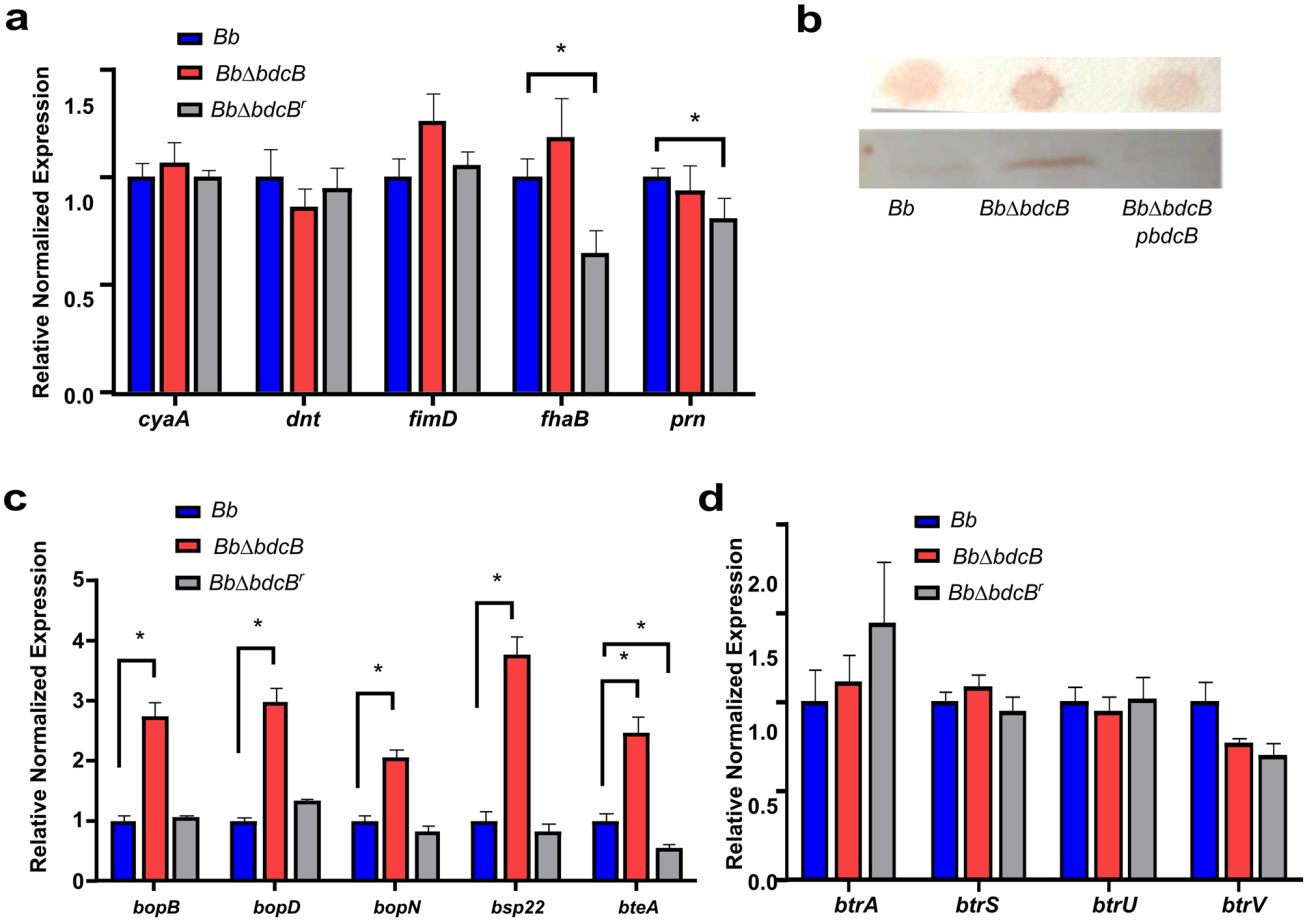
BdcB regulates T3SS expression. (**A**) Virulence factors mRNA amounts were determined by quantitative real-time PCR. Fold changes were calculated by the ΔΔCT method using *recA* levels as a control. Three biologically independent experiments with three technical replicates were performed. Mean fold changes of triplicate cultures were compared using Student’s paired t-test (two-tailed distribution) *,* p *< 0.01. (**B**) Cells surface and supernatant levels of Bsp22 were measured by dot blot and western blot respectively. Shown is a representative dot blot and western blot of two assays in *Bb*, *Bb*Δ*bdcB,* and *Bb*p*bdcB* strains. (**C**, **D**) T3SS components and *btr* regulon mRNA amounts were determined by quantitative real-time PCR. Fold changes were calculated by the ΔΔCT method using *recA* levels as a control. Mean fold changes of triplicate cultures were compared using Student’s paired t-test (two-tailed distribution), *,* p *< 0.01.

Following up with our observations of increased cytotoxicity and greater proinflammatory responses, we decided to investigate the expression of virulence factors of the three *B. bronchiseptica* strains. *B. bronchiseptica* contains different virulence factors that contribute to the suppression of immune responses. We hypothesized that BdcB may regulate the expression or activity of these proteins through the regulation of c-di-GMP levels. Adhesins like filamentous hemagglutinin (FHA), fimbriae, and pertactin have been associated with immune regulatory activities^33^. Also, the RTX toxin adenylate cyclase (ACT) has been shown to inhibit monocytes maturation, presents pro-apoptotic activity in phagocytes, and enhances IL-10 and IL-17 production by CD8 + T cells^34^. Our results did not reveal differences in the mRNA levels of FHA, ACT or other virulence factors analyzed (Fig. 7A). Unexpectedly, we observed significative differences with the reconstituted strain in *fhaB* and *prn* expression.

Based on the immune response observed in this work, we hypothesized that BdcB might have an impact on the levels of expression of the T3SS, which could explain the increased cytotoxicity identified in vitro as well as the observed cytotoxicity and cytokine profiling. T3SS system is required for *B. bronchiseptica* cytotoxicity on different eukaryotic cells (macrophage cells, dendritic cells, etc.) and has also been shown to be responsible for the induction of apoptosis in macrophages^21,35^. In addition, our previous work showed that c-di-GMP regulates T3SS expression^17^. We first analyzed Bsp22 protein production. This protein is part of the needle of the system and is the most abundant protein exported by the T3SS in *B. bronchiseptica*. We performed a dot blot assay to analyze the amount of protein on the bacterial surface and a western blot to analyze the presence in the supernatant (Fig. 7B and Fig. S9). In addition to analyzing the *Bb* and *Bb*Δ*bdcB* strains, we used the *Bb-*p*bdcB* strain that overexpresses *bdcB*. Our results revealed that higher amounts of Bsp22 were found in *Bb*Δ*bdcB* compared to the *Bb*, while the *Bb-*p*bdcB* strain presented a lower production than *Bb*. The control results were consistent with our previous work^17^. These results showed that in the absence of *bdcB,* production of the T3SS needle increased, and contrary, overexpression of *bdcB* resulted in a decrease in the production of the needle protein Bsp22.

We then analyzed the expression of T3SS at the transcriptional level by qRT-PCR (Fig. 7C). We evaluated five components of this system: BopB and BopD, which can interact and form pores in the plasma membrane of the host cell to allow the passage of effector proteins; BteA, which encodes for one of the effector proteins of the system; BopN that functions as a “stopper” to prevent secretion until the right conditions are met (contact with the host cell or certain in vitro conditions) and Bsp22 itself (Fig. 7C). As expected, based on our previous results, *Bb*Δ*bdcB* had higher expression of *bsp22*. Also, the expression of *bopN*, *bopB*, *bopD,* and *bteA* was increased in the mutant, indicating that in the absence of *bdcB* several components of the T3SS were upregulated.

Genes regulated by BdcB are part of the *bsc* regulon^20^ that is regulated by the transcriptional factors coded by the *btr* regulon. To investigate if *bdcB* has an impact on the *btr* regulatory system, we analyzed the *btr* regulon (*btrS*, *btrA*, *btrU* and *btrV*). We determined the mRNA levels of *btrS*, *btrA*, *btrU,* and *brtV* using qRT-PCR (Fig. 7D). Our results revealed that there were no significant differences between the mRNA levels of the three bacterial strains in any of the four genes encompassed in the *brt* regulon analyzed. Although no differences were observed at the transcriptional level in the *btr* regulon, we cannot rule out that BdcB acts on these through some post-transcriptional mechanism or that another regulatory mechanism exists that we do not know yet.

## Discussion

*Bordetella* spp. harbor many mechanisms that allow them to successfully colonize the host and transmit to a new one. However, these virulence mechanisms require a fine-tuning gene regulation that as in other species, also involves c-di-GMP. Active DGC dimerizes to combine two GTP into one molecule of c-di-GMP. Dimerization is facilitated by the N-term domain, frequently a signal response domain. In the present work, we described the *Bordetella* diguanylate cyclase BdcB and its impact on host immunomodulation. Analysis of the primary structure and our experiments strongly suggest a DGC activity for this protein. As expected for an active DGC, and described previously by us in *B. bronchiseptica* for other DGC, constitutive expression of *bdcB* from a plasmid inhibits motility and enhances biofilm formation in *B. bronchiseptica*^10^. Is important to note that we did not expect an exaggerated biofilm production when *bdcB* was expressed. Biofilm formation by *B. bronchiseptica* is high in intermediate phase and probably is close to the maximum that bacteria can achieve. Over expression of a DGC might only push slightly more biofilm, as we observed before with other DGCs^10^.

The predicted structure of BdcB is composed of a complete GGDEF domain but not a well-defined sensor domain in the N-term portion. The first 46 aminoacids in the N-term domain present an intrinsically disordered region (IDR). These regions are unstructured regions with no clearly defined role. However, some IDR have been associated with protein–protein or ligand–protein interactions^26^. Interestingly, the IDR region was important for BdcB function. Overexpression of the truncated version of *bdcB* (Δ*NbdcB*) was not able to inhibit motility nor enhanced biofilm formation. It has been described that dimerization in DGCs is mediated by N-term domains and necessary for DGC activity, therefore we speculate that the IDR region may be involved in the dimerization process and correct orientation of the GGDEF domain. Moreover, since some IDRs adopt a defined structure when a ligand binds to them, we propose that dimerization by the IDR region might be stimulated by an unknown intracellular ligand or other unknown protein.

Sloan and co-workers reported that defective biofilm formation in vitro correlates with lower levels of bacteria in the nasal cavities of infected mice^36^. The presence of a polysaccharide synthesized by the *bps* locus is important for biofilm formation in vitro and in vivo^28,37^. Due to the difference in biofilm formation by *Bb*Δ*bdcB* that we observed, we anticipated differences from *Bb* colonization in the mouse model. However, in two independent mouse models, we could not observe differences in colonization between strains when infecting at high dose high volume. Moreover, our infection model lasted for 14 days, and we did not assess if longer term persistence due to enhanced ability to form biofilm could impact the chronicity of the disease. Excitingly, cytokines and chemokines profiling were different, and the lungs of mice infected with *Bb*Δ*bdcB* presented a more enhanced immune response, characterized by chemokines that recruit neutrophils to the lung to limit the infection. Of note, this enhanced immune response was not sufficient to clear the mutant infection during the time we followed the experiment, suggesting *B. bronchiseptica* is resistant enough to avoid the exacerbated immune response.

Bacteria can survive inside eukaryotic cells by different mechanisms. Particularly inside macrophages, resistance to acidic pH is a mechanism usually observed in pathogens with an intracellular lifestyle. Accordingly, *Bb*Δ*bdcB* did not survive to acid pH (pH 4.5) like wild type *Bb*. Moreover, overexpression of *bdcB*, and in consequence high levels of c-di-GMP protect bacteria from acidic stress. Protection obtained by BdcB was not specific, since other active DGC, BdcA, also protected bacteria against low pH showing that high c-di-GMP concentration is the signal for induction of response to acidic stress. Although BdcB may be involved in resistance to acidic pH present within macrophages, *bdcB* was not significantly more expressed inside BMM. DGCs are usually activated by allosteric binding to accessory domains. Activation of BdcB activity, without overexpressing *bdcB*, can be explained if a ligand stimulates BdcB activity inside BMM. We speculate that the nature of the ligand might be related to pH descendant.

Due to the differences in the cytokine profiling, we investigated the effects of *bdcB* in host cells using bone marrow-derived macrophages. Our results suggests that *bdcB* is required for long-term survival in macrophages. We observed enhanced macrophage death, combined with a greater susceptibility to acidic pH due to the absence of this gene, indicating the importance of BdcB for intracellular survival. Interestingly, the macrophage´s response to infection mimics what we have found in vivo: pro-inflammatory cytokines were increased, and cell death was also enhanced. Notably, *Bb*Δ*bdcB* induced a higher inflammatory response compared to *Bb*-infected BMM. Cytokines such as TNF-alpha, IL-6, and IL-10 were significantly enhanced by the mutant. These cytokines are a key component of the immune response to *Bordetella*^33,38^. Considering the absence of viable bacteria and the enhanced proinflammatory signals we speculate that bacteria might lysate the BMM. Indeed, we observed that *Bb*Δ*bdcB* induced cell death.

The stimulation of pro- and anti-inflammatory cytokines in *Bb*Δ*bdcB*- infected mice may seem contradictory. However, the absence of BdcB during the infectious process would prevent a necessary BdcB-mediated regulation of c-di-GMP. If a variable c-di-GMP concentration is needed for an effective infection and persistence, the *Bb*Δ*bdcB* will fail to control those fluctuations and we will observe conflicting signals produced by immune cells. Overall, we evidenced that regulation of c-di-GMP levels during infection may not be detrimental for the number of bacteria established in the respiratory tract but is important to regulate the immune response.

The regulation of T3SS by the second messenger c-di-GMP has been described in other pathogens and *B. bronchiseptica*^17,39^. The T3SS has been already analyzed in *Bordetella*: it is responsible for the necrosis of macrophages and induction of IL-10. BteA, one of the T3SS effectors has been described as a potent cytotoxin, responsible for necrosis induction of eukaryotic cells^40^. Here we showed that BdcB is involved in the regulation of the *bsc* locus in a mechanism where expression of other known T3SS regulators such as BtrS or BtrA are not involved. Interestingly, *bdcB* expression was not affected by intracellular lifestyle. Post-transcriptional regulation involving contact between proteins may explain BdcB activity regulation. Participation of the IDR region of BdcB may be considered to explain the activity regulation of this DGC.

Overall, we described in this work a unique DGC, with an IDR region, essential for DGC activity, that regulates the T3SS in *B. bronchiseptica* that could be shared with other *Bordetellae*. The T3SS is a virulence factor tightly regulated by different mechanisms and important for *Bordetella* pathogenesis. Also, we demonstrated that the second messenger c-di-GMP is involved in the regulation of the infection process, particularly in the intracellular lifestyle.

## Material and methods

### Protein structure prediction

The protein structure was predicted using ColabFold^23^. Sequence alignments were generated through MMseas2 and HHsearch. All results zip files are available as ﻿supplemental material.

### Bacterial strains and growth conditions

*B. bronchiseptica* 9.73H + was isolated from a rabbit and described previously^41^. *B. bronchiseptica* isolates were grown on Bordet-Gengou agar (BGA) (Difco) supplemented with 15% (vol/vol) defibrinated fresh sheep blood at 37 °C for 48 h. Stainer–Scholte (SS) liquid medium was used to grow *B. bronchiseptica* in broth cultures^42^. If necessary, BGA or SS were supplemented with gentamicin (50 µg ml^-1^), kanamycin (80 µg ml^-1^), and/or streptomycin (200 µg ml^-1^).

*P. fluorescens* strains were grown in lysogeny broth (LB) at 30 °C with 30 μg ml^-1^ gentamycin.

### Plasmid and strain construction

Plasmids and strains were constructed using standard molecular biology techniques; detailed descriptions of plasmid and strain construction procedures are in the supplemental material (Text S1).

### Biofilm formation assays

*P. fluorescens* and *B. bronchiseptica* biofilm assays were performed as previously described^11^. Briefly, for *B. bronchiseptica* assays, we selected hemolytic colonies grown in SS semisolid medium (1.5% agar) supplemented with 15% (vol/vol) defibrinated fresh sheep blood. Bacteria were resuspended in SS medium and pipetted into wells of a sterile 96-well U-bottom microtiter plate (polyvinyl chloride [PVC]). Nicotinic acid was added at the indicated concentrations when appropriate. Biofilms were grown statically for 24 h at 37 °C. For *P. fluorescens* assays, the overnight culture was inoculated into K10T-1 medium at final OD_600_:0.1 and 100 µl of this suspension was transferred into a 96-well U-bottom microtiter plate (polyvinyl chloride [PVC]) and grown statically for 6 h at 28 °C.

In both assays, after incubation planktonic bacteria were removed and attached cells were stained with 0.1 g/l crystal violet (CV) solution. The stain was dissolved by adding 120 ml 33% acetic acid solution and then quantified by measuring OD595^10^. Experiments were repeated at least three times with at least four technical replicates.

### Scanning electron microscopy

*B. bronchiseptica* biofilm assays for scanning electron microscopy were performed as previously described by our group^7,8^. Bacteria were cultured in SS medium over glass coverslips vertically submerged in plastic tubes for 24 h. Coverslips were then treated to perform a CO_2_-critical-point procedure (EmiTech K850) and sputter coated with gold. Samples were visualized with a scanning electron microscope (FEI Quanta 200), and the images were processed with Image Soft Imaging System ADDA II. At least two independent samples were analyzed per strain. Most of the sample was scanned and representative images were selected for processing.

### Motility assays

SS soft agar motility plate (0.35% agar) supplemented with 40 mM MgSO_4_ was used to determine the motility of bacterial strains as previously described^10^. The diameter of the migration zone was measured after 18 h of incubation at 37 °C. Experiments were repeated at least three times with at least three technical replicates.

### SDS PAGE and Western blot analysis

For HA tag detection, *B. bronchiseptica* cells were collected, normalized to an equal OD_650_ value, harvested by boiling samples in 1X Laemmli buffer for 10 min, and subjected to SDS–polyacrylamide gel electrophoresis followed by transfer of the contents of the gel to a polyvinylidene fluoride (PVDF) filter. To detect Bsp22 supernatants of mid-logarithm liquid culture were collected, filtered through a low-binding protein filter with a 0.22-mm pore size and concentrated with Amicon Ultra centrifugal filter (molecular weight cutoff [MWCO] 10 kDa). Samples were normalized according to culture OD_650_ and subjected to SDS–polyacrylamide gel electrophoresis followed by transfer of the contents of the gel to a polyvinylidene fluoride (PVDF) filter. For dot blot analysis, cells were collected, normalized to an equal OD_650_ value, washed with phosphate buffer, and resuspended in 500 µl of phosphate buffer. 20 µl of the suspension was deposited onto the filter and left at room temperature until dried.

The PVDF membrane (for western blot or dot blot) was blocked for 1 h followed by incubation with indicated polyclonal serum diluted 1:2000 in TBS containing 5% nonfat milk powder at 4 °C overnight. The filter was then incubated with anti-mouse IgG conjugated to horseradish peroxidase (HRP) (1:3000) (Invitrogen) in TBS containing 5% nonfat milk powder at room temperature for 2.5 h. Horseradish peroxidase-conjugated anti-mouse antibody (Bio-Rad, USA) was used as the secondary antibody. A chemiluminescent reagent or DAB (3,3′-diaminobenzidine) reagent was used for developing according to the manufacturer’s instructions. Samples from each strain and growth condition combination were prepared and analyzed independently three times.

### Animal experiments

All animal experiments in this article were conducted in accordance with the ARRIVE guidelines. Wild type C57BL/6 J and BALB/c mice were purchased from Jackson Laboratories, Bar Harbor, ME or our breeding colony (established from Jackson Laboratories mice). Breeding colonies were maintained under the care of the employees and veterinarians of Louisiana State University Health—Shreveport Animal Care Facility, Shreveport, LA, (AUP:20-038, AUP:22-031). LSU Health Shreveport animal facilities is AAALAC-accredited and assurance with the Office of Laboratory Welfare, and National Institute of Health. Facility is staffed with three full-time veterinarians. All our animal experiments were performed accordingly with our approved protocols (Louisiana State University Health Shreveport IACUC Protocol number P20-038 and P22-031 that include the animal procedure protocol and breeding protocol. All the experiments approved by our IACUC assure animal welfare and humane endpoints).

Four to Six-week-old BALB/c or C57BL/6 J mice were used as a model for in vivo respiratory infection by *B. bronchiseptica*. Bacteria were grown overnight on SS medium at 37 °C and were diluted in PBS to provide the challenge doses. For inoculation, mice were anesthetized with 5% isoflurane and were inoculated by pipetting directly into each nostril with 30 μL of 1 × 10^6^ CFU/mL. The inoculum was confirmed by plating dilutions on BGA and counting colonies after incubation for two days at 37 °C. At indicated days post-challenge, five (BALB/c) or four (C57BL/6 J) mice from each group were euthanized. Mice were euthanized using 5% CO_2_ followed by cervical dislocation. Following euthanasia, the nasal cavity, trachea, and lungs were collected in 1 mL of cold PBS in 2 ml tissue homogenization tubes containing a mixture of 0.5 mm and 1.4 mm ceramic beads. Tissues were homogenized, serially diluted, and plated on BGA. Colonies were enumerated after incubation at 37 °C for two days. Data form BALB/c experiments were pooled from two independent experiments.

### Quantification of cytokines and chemokines

The cytokine and chemokine responses were evaluated using LEGENDplex™ (BioLegend) bead-based immunoassays. Undiluted nasal cavity and lung homogenates were centrifuged at 10,000 g for 10 min. The supernatants of lung homogenates and the supernatants of infected macrophages were analyzed as per the manufacturer’s instructions for using serum/plasma. For the cytokine analysis of cell culture supernatants, the T-helper (Th) cytokine panel version 2 and the inflammation panel were used. For the analysis of chemokines and cytokines in the nasal cavity and the lungs of mice, the proinflammatory chemokine panels and the inflammation panel were used. Once the plate was ready, samples were analyzed using the NovoCyte Quanteon cytometer in Immunophenotyping core at LSU Health Science Center, Shreveport. For data analysis we used the software recommended “legendplex.qognit” using as standard the amount provided in each kit. Heatmaps were drawn from the normalization of each cytokine/chemokine with respect to the maximum value measured. To normalize the data, we use the MinMaxScaler class from the Python scikit-learn library. To make the plots we use the matplotlib and Seaborn libraries, both from Python.

### Isolation of bone marrow-derived macrophages

Bone marrow-derived macrophages were prepared by culturing cells harvested from the femur of BALB/c mice in DMEM (Sigma) supplemented with 10% v/v fetal bovine serum (FBS). L929 cell supernatants were used as a source of macrophage colony-stimulating factors and were added to the medium at 20% v/v of the total volume. Macrophages were cultured for 7 days at 37 °C in a 5% CO_2_ atmosphere, with the fresh medium being changed every 3 days^43^.

### Cytotoxicity assay

Bone marrow-derived macrophages were cultured in DMEM medium supplemented with 10%v/v fetal bovine serum (FBS) and 20% v/v of L929 cell supernatants to 90% confluency. The macrophages were washed twice with PBS Gibco™, pH 7.2 from Thermo Fisher Scientific. *B. bronchiseptica* strains grown on BGA plates were resuspended in RPMI medium supplemented with 10% v/v fetal bovine serum (FBS). The bacteria were added to the macrophages at MOI 1, 10, and 100, and the samples were centrifuged at 300 g for 10 min. The preparations were incubated for 4 h at 37 °C in a 5% CO_2_ atmosphere. The cytotoxicity was measured as the release of LDH using the CytoTox 96 Kit (Promega) following the manufacturer’s protocol.

### pH resistance assays

Bacterial suspensions from an overnight culture were obtained at approximate 3 × 10^8^ bacteria/ml in SS medium. This suspension was then used to inoculate three tubes, containing SS medium pH:4.0 supplemented with gentamicin, in a final concentration of 3 × 10^6^ bacteria/ml of each strain. To diminish the pH values of SS medium pH 4.0, the Tris–HCl buffer was replaced by MES (2-(N-morpholino) ethane-sulfonic acid (20 mM))^44^. Strains were tested simultaneously. Each of these tubes corresponds to a technical replicate. Once the inoculation was done, the suspension was homogenized, and an aliquot was quickly taken and placed in PBS to perform the initial bacterial count. The bacterial suspensions were then incubated for 4 h at 37 °C under agitation. After the incubation time, the final sample was taken to make the final count. The samples were serially diluted in sterile phosphate saline buffer (PBS) and 10 μl aliquots were plated in BGA. Viable colonies recovered after an incubation of 48 h at 37 °C were counted. With the initial and final count of each of the tested strains the number of surviving bacteria was calculated and normalized relative to the wild-type strain. At least three independent assays with duplicates for each strain were performed.

### Macrophage propidium iodide assay

Bone marrow-derived macrophages were cultured in DMEM medium supplemented with 10% v/v fetal bovine serum (FBS) and 20% v/v of L929 cell supernatants to 90% confluency. The macrophages were washed twice with PBS Gibco™, pH 7.2 from Thermo Fisher scientific. *B. bronchiseptica* strains grown on BGA plates were resuspended in RPMI medium supplemented with 10% v/v fetal bovine serum (FBS), 25 mM HEPES, and 10 μg/ml of propidium iodide (Invitrogen, cat number P1304MP). Bacteria were added to the macrophages at MOI 1 and the samples were centrifuged at 300 g for 10 min. The preparations were incubated with a humidity chamber in the Spark TECAN multimode plate reader overnight. Fluorescence measurements were performed every 10 min for 8 h at 37 °C. (Excitation Length: 535 nm and Emission Length: 624 nm).

### Intracellular survival assay

Bone marrow-derived macrophages were cultured in DMEM medium (GIBCO) supplemented with 10% v/v fetal bovine serum (FBS) (GIBCO) and 20% v/v of L929 cell supernatants to 90% confluency at 37 °C in a 5% CO_2_ atmosphere. The macrophages were washed twice with PBS Gibco™, pH 7.2 from Thermo Fisher scientific. *B. bronchiseptica* strains grown on BGA plates were resuspended in DMEM medium supplemented with 10% v/v fetal bovine serum (FBS) and 20% v/v of L929 cell supernatants. The bacteria were added to the macrophages at MOI: 100, and the samples were centrifuged at 300 g for 5 min and incubated at 37 °C. After 1 h, 100 μL of 0.1% Triton X-100 solution (Thermo-scientific, VWR cat number AAA16046-AE) in PBS was administered to a subset of wells, followed by a 5-min incubation at room temperature and vigorous pipetting to lyse open cells. 10 μL dilutions were serially diluted in sterile PBS and plated on a BGA medium to quantify the total bacteria (intracellular and extracellular) present after 1 h. At one hour, the supernatant was removed from the remaining wells and replaced with 100 μL of 100 μg/mL gentamicin solution (Sigma-Aldrich, cat number 1405-41-0) in DMEM to the remaining sample wells. Plates were incubated in 5% CO_2_ at 37 °C, and then at 2- and 4 h post-gentamicin addition, appropriate wells were washed 3 × with DMEM and treated with 100 μL 0.1% Triton X-100 as described above to determine the number of total bacteria.

### RNA isolation and quantitative real-time PCR

Three independent biological replicates of *B. bronchiseptica* strains were grown in SS medium at 37 °C with shaking to the exponential growth phase at an optical density of OD_600_ = 0.7. The experiments were performed in independent triplicates. Bacteria were harvested by centrifugation at 6000 g for 5 min. Total RNA was extracted from the bacteria using the RNAeasy Kit (Qiagen, Valencia, CA, United States), and treated with RNase-free DNase I (Invitrogen, Carlsbad, CA, United States) according to the manufacturer’s instructions. After the extraction, the RNA solutions obtained were quantified using the Thermo Fisher NanoDrop™ One/One^C^ Microvolume UV–Vis Spectrophotometer. To carry out the qRT-PCR assays, the Luna^R^ Universal one-step RT-qPCR kit (New England Biolab) was used. This kit converts RNA to cDNA and subsequent cDNA amplification using a single reaction mix. The primers used are listed in Supplemental Table 1. The procedures were performed according to the manufacturer's instructions using 5 μl of 25 ng/μl RNA in each reaction mixture. The reaction was run in the CFX96 Bio-Rad thermocycler, and the protocol was recommended by the manufacturer. Briefly, 55 °C 10 min, followed by initial denaturalization 95 °C 1 min, followed by 45 cycles of 95 °C 10 s, annealing 15 s, an extension of 60 °C 30 s, and finally a final extension of 60 °C 10 min.

Three biological replicates and three technical replicates were performed for each gene analyzed. The *recA* gene was used as the reference gene since it is constitutively expressed in *B. bronchiseptica*. Expression levels were calculated using the Bio-Rad CFX96 Maestro program.

### Statistical analysis

At least two biological replicates were performed in each experiment (the number is indicated in each experiment). Means were analyzed for significance using a one-way ANOVA with a Tukey’s multiple-comparison test to compare differences among groups. The significance level is stated in each figure legend.

### Accordance statement

All methods were performed in accordance with the relevant guidelines and regulations.

## Supplementary Information

Supplementary Information 1.

Supplementary Information 2.

## Data Availability

The GenBank accession number for the *B. bronchiseptica* RB50 genome is NC_002927.3. Gene identification number and locus tag, respectively, for *bdcB* are 2,661,408 and BB_RS19575. The datasets generated during and/or analyzed during the current study are available in GenBank repository, https://www.ncbi.nlm.nih.gov/genome/?term=NC_002927.3 (NC_002927.3) and https://www.ncbi.nlm.nih.gov/nuccore/33598993 (BB_RS19575).
