# Supplementary material for: Bordetella bronchiseptica diguanylate cyclase BdcB inhibits the type three secretion system and impacts the immune response
Source: Sci Rep. 2023 May 2;13:7157. doi: 10.1038/s41598-023-34106-x (PMC10154355; doi:10.1038/s41598-023-34106-x)
Supplement: Supplementary file 1 — Supplementary Information 1. [file 41598_2023_34106_MOESM1_ESM.pdf]

Supplementary file Belhart *et al.*

Detailed Materials and Methods

Strains and media.

Escherichia coli (DH5α and S17-1) strains were cultured with lysogeny broth (LB) in a test tube or on solidified LB with 1.5% agar. When appropriate, antibiotics were added to the medium at the following concentrations: 10 μg ml^{-1} gentamicin and 50 μg ml^{-1} kanamycin.
Replicative plasmids were introduced to *E. coli* by electroporation using standard techniques. Non- and replicating plasmids were introduced into *Bordetella bronchiseptica* by conjugation. The yeast strain InvSc1 (*Saccharomyces cerevisiae*; Invitrogen), was routinely cultured on YPD medium. When selecting for plasmids carrying the URA3 gene, yeast was grown on YNB with complete supplemental mixture minus uracil.

Yeast cloning for plasmid construction:

All oligonucleotide primers used in the study are listed below. Cloning was performed by *in vivo* recombination in yeast, as described[1]. Briefly, vectors bearing the 2μ replication origin and URA3 gene were linearized by restriction enzyme digestion. One or more inserts were generated by PCR using primers with 30 or more non-annealing bases to facilitate homologous recombination with adjacent fragments and/or the vector. Then, PCR fragments and linearized vector were introduced into yeast by transformation and recombinant vector bearing insert(s) of interest were selected for on uracil minus medium. All PCR was performed using Phusion polymerase (NEB).

Plasmid construction

Plasmid based on the pMQ30 allelic replacement vector was used to generate Δ*bdcB* knockout strains in wild type background. Two stretches of homologous DNA flanking the genomic region to be deleted were amplified by PCR (430-340 bp each), utilizing primers with 30 or more extra bases to facilitate recombination with adjacent fragments in cloning. *nptII* gene was amplified from the pKD13 plasmid. Yeast cloning techniques were employed to introduce the three fragments in the pMQ30 plasmid to yield pMQ30F1KmF2. Plasmid recovered from yeast was electroporated in *E. coli* S17-1.

Plasmid based on the pMQ30 allelic replacement vector was used to reconstitute the Δ*bdcB* knockout strain to wild type. The *bdcB* and 200 pb upstream and downstream were amplified by PCR with primers listed below with 30 or more extra bases to facilitate recombination with adjacent fragments in cloning. Yeast cloning techniques were employed to introduce the fragment in the pMQ30 plasmid. Plasmid recovered from yeast was electroporated in *E. coli* S17-1.

Plasmids based on the pMQ72 vector were used to over-express *bdcB* gene and variants (ΔN and GGAAF) genes. The gene of interest was amplified by PCR with primers listed below with 30 or more extra bases to facilitate recombination with adjacent fragments in cloning. Yeast cloning techniques were employed to introduce the fragment in the pMQ72 plasmid. Plasmid recovered from yeast was electroporated in *E. coli* S17-1. The *bdcB* gene and variants (ΔN and GGAAF) were subcloned by classical techniques to broad-host range plasmid pBBR1MCS-5 with a constitutive promoter *npt*II[3].

All constructions were verified by PCR and DNA sequencing.

<u>Allelic replacement</u>

Briefly, constructs were introduced into *B. bronchiseptica* by conjugation with *E. coli*, and transconjugants selected on BGA 80 µg ml^{-1} kanamycin and 200 µg ml^{-1} streptomycin. Colonies were cultured O/N with 80 µg ml^{-1} kanamycin, then plated on Stainer Scholte media and selected Km^R and Gm^S for possible *bdcB* mutants. Mutants were verified by PCR and sequencing of purified genomic DNA to ensure proper construction.

<u>Construction of strains with vectors</u>

Constructs were introduced into *Pseudomonas fluorescens* by electroporation as described before and transconjugants were selected on LB 30 µg ml^{-1} gentamicin[2].

Constructs were introduced into *B. bronchiseptica* by conjugation with *E. coli*, and transconjugants selected on BGA 80 µg ml^{-1} gentamicin and 200 µg ml^{-1} streptomycin. For detailed protocol of *B. bronchiseptica* conjugation see 7.

<u>RNA isolation and Quantitative Real-Time PCR</u>

Three independent biological replicates of *B. bronchiseptica* strains were grown in SS medium supplemented with nicotinic acid at 37°C with shaking to the exponential growth phase at an optical density of OD600 = 0.7. The experiments were performed in independent triplicates. Bacteria were harvested by centrifugation at 6000 g for 5 min. Total RNA was extracted from the bacteria using the RNAeasy Kit (Qiagen, Valencia, CA, United States), and treated with RNase-free DNase I (Invitrogen, Carlsbad, CA, United States) according to the manufacturer’s instructions. After the extraction, the RNA solutions obtained were quantified using the Thermo Fisher NanoDrop™ One/OneC Microvolume UV-Vis Spectrophotometer. To carry out the qRT-PCR assays, the LunaR Universal one-step RT-qPCR kit (New England Biolab) was used. This kit converts RNA to cDNA and subsequent cDNA amplification using a single reaction mix. The primers used are listed in Supplemental Text 1. The procedures were performed according to the manufacturer's instructions using 5 µl of 25 ng/µl RNA in each reaction mixture. The reaction was run in the CFX96 Bio-Rad thermocycler, and the protocol was recommended by the manufacturer.

Briefly, 55 °C 10 minutes, followed by initial denaturalization 95 °C 1 minute, followed by 45 cycles of 95 °C 10 seconds, annealing 15 seconds, an extension of 60 °C 30 seconds, and finally a final extension of 60 °C 10 minutes. Three biological replicates and three technical replicates were performed for each gene analyzed. The *recA* gene was used as the reference gene since it is constitutively expressed in *B. bronchiseptica*. Expression levels were calculated using the Bio-Rad CFX96 Maestro program.

Table S1. Plasmids used in this work

Plasmids	Description	Reference
pMQ72	*Pseudomonas* expression vector, GmR	[1]
pMQ72-*bdcB*	pMQ72 containing *bdcB* fused to HA	This work
pMQ72-*gcbC*	pMQ72 with *gcbC* from *P. fluorescens*	[2]
pMQ72-*bdcB* (GGAAF)	pMQ72 with *bdcB* with GGDEF site modified by GGAAF and fused to HA	This work
pMQ72-ΔN-*bdcB*	Vector pMQ72 with the *bdcB* missing the first 165 nucleotides and fused to HA	This work
pBBR1MCS-5-*npt*II	pBBR1MCS-5 with *npt*II promotor	[3]
p*bdcB*	pBBR1MCS-5-*npt*II with *bdcB* fused to HA downstream promotor nptII	This work
p*bdcA*	pBBR1MCS-5-nptII with *bdcA* downstream promotor nptII	[3]
pbdcB (GGAAF)	pBBR1MCS-5-nptII with *bdcB* with GGDEF site modified by GGAAF and fused to HA downstream promotor nptII	This work
pΔN-bdcB	pBBR1MCS-5-nptII with *bdcB* missing the first 165 nucleotides and fused to HA downstream promotor nptII	This work
pMQ30	allelic replacement; *sacB aacC1* ColE1 *oriT* CEN4 URA3	[1]
pMQ30F1KmF2	pMQ30 containing *bdcB* upstream and downstream region separated by a kanamycin resistance gene.	This work
pMQ30*bdcB*	pMQ30 containing *bdcB* with its promotor	This work

Table S2. Strains used in this work

Strains	Description	Reference
PsΔ4	*Pseudomonas fluorescens* Pf0-1 with an unmarked deletion of *wspR, gcbA, gcbB* and *gcbC*	[2]
PsΔ4-pEmpty	PsΔ4 with pMQ72	[2]
PsΔ4-p*gcbC*	PsΔ4 with pMQ72-*gcbC*	[2]
PsΔ4-p*bdcB*	PsΔ4 with pMQ72-*bdcB*	This work
PsΔ4-*pbdcB* (GGAAF)	PsΔ4 with pMQ72-*bdcB* (GGAAF)	This work
PsΔ4-pΔN-bdcB	PsΔ4 with pMQ72-ΔN-*bdcB*	This work
Bb	Wild type strain; 9.73H+ Sm^r	[4]
Bb-pEmpty	Bb9.73H+ wild-type strain with pBBR1-MCS-5-*npt*II	[3]
Bb-p*BdcB*	Bb9.73H+ wild-type strain with p*bdcB*	This work
Bb-p*bdcB* (GGAAF)	Bb9.73H+ wild-type strain with p*bdcB* (GGAAF)	This work
Bb-pΔN-bdcB	Bb9.73H+ wild-type strain with p-ΔN-*bdcB*	This work
BbΔ*bdcB*	Bb9.73H+ with *bdcB* disrupted with a kanamycin resistance gene	This work
BbΔ*bdcB*^{r}	BbΔ*bdcB* reconstituted with *bdcB* with its promoter	This work
BbΔ*bdcB*-pEmpty	BbΔ*bdcB* with pBBR1-MCS5-pnptII	This work

Bb-p*BdcA*	Bb9.73H+ wild-type strain with p*bdcA*	[3]

Table S3. Primers for qRT-PCR

ORF	Name/ Annotation	Forward Primer	Reverse Primer	Reference
BB4228	bteA	CGTGAAATGGCTGGATGG	GCGTCGGTGGATTGCTG	[5]
BB0324	cyaA	CACTGAGCAGAACAATCCTTTCC	CGTGAGCATCTGGCTTTCAC	[5]
BB1366	prn	CAGCACGGCATCCACATC	GCCTGACGACCGCTTACC	[5]
BB1616	bopN	TGCCGAGGAAAAGCATCACT	GCCAGAGCATCGGACGTT	[5]
BB1617	bsp22	CGGCACGGGCGTCAT	GGTGTAGGCACTTTCGAGTTCCT	[5]
BB1620	bopD	CGGCTCGGTGAAGACATCTAC	GCCTCCCGCATCTGTTGA	[5]
BB1621	bopB	GCTCAATTCGACGAGGCCTAT	TGTGCGTACTCGCCATATCG	[5]
BB2989	fimD	GTATCCCCAGAAAGGCGAAGT	GCGAACCTCCGCGTTGT	[5]
BB2993	fhaB	GGAATCAGTGCCGACTTCGA	AGTTCCCACCCAGATATTGGGTAT	[5]
BB3978	dnt	GCAGAAAGTACGGCACTACAAGGT	CCTGTTGTGATTTTCGATTCCA	[5]
BB1638	btrS	CACTGCTTTCGGTTCCTGT	ACGCTGTCGTAGCCTCGTT	[6]
BB1639	btrA	CCGCTTTACCTGCTTTGGAG	GATGCGATGACCTGGAACTG	[6]
BB1646	btrV	GCGGCTTGACTACATTTCCA	TTCGCACAGCACCAGTT	[6]
BB1642	btrU	TACGAGGAAAGCCGCAAC	GAGATGGCGAGAACCCACTT	[6]
BB3903	bdcB	GATCAGCGCAAGTCCTCG	TTCGGCGCGCAGGTAATT	This work

Table S4. Primers to build *Bb∆bdcB* y *Bb∆bdcB*^{r}

Name/ Annotation	Primer	Reference
F1BB3903Fb	CTGTTTTATCAGACCGCTTCTGCGTTCTGATAATCCCATCGCCTGCT	This work
F1BB3903R	TCTAGAAAGTATAGGAACTTCGAAGCAGCTCCAGCCTAGCGTTGTCCAGCCTCG	This work
F2BB3903F	AGGTCGACGGATCCCCGGAATTAATTCTCATGTTTCATAACGGCCCGCGG	This work
F2BB3903Rb	CAATTTCACACAGGAAACAGCTATGGTGGCAGTATGCGCGC	This work

Table S5. Primers to build p*bdcB* /p*bdcB*-(GGAAF) /p-ΔN-*bdcB*

Name/ Annotation	Primer	Reference
PMQ3903F1	GCGAATTCGAGCTCGGTACCCGGAGGGATTGC GATGGCGAGGCTG	This work
GGAAF3903R1	GACAGCACCGCGGCGAACGCCGCGCCGCCCA CCC	This work
GGAAF3903F2	GGGCGGCGCGGCGTTCGCCGCGGTGCTGTCG	This work
ΔN55BB3903F	GCGAATTCGAGCTCGGTACCCGGGATTATATG GGCGCCGCGACGCTGCAGG	This work
reverse3903HA	GAACATCGTATGGGTATGCGCCAGCCGTGCGG CTGG	This work
F HA 3903	CACGGCTGGCGCATACCCATACGATGTTCCTG ACTATGC	This work
R HA 3903	AGGTCGACTCTAGAGGATCCCCTTAAGCGTAA TCTGGAACGTCATA	This work

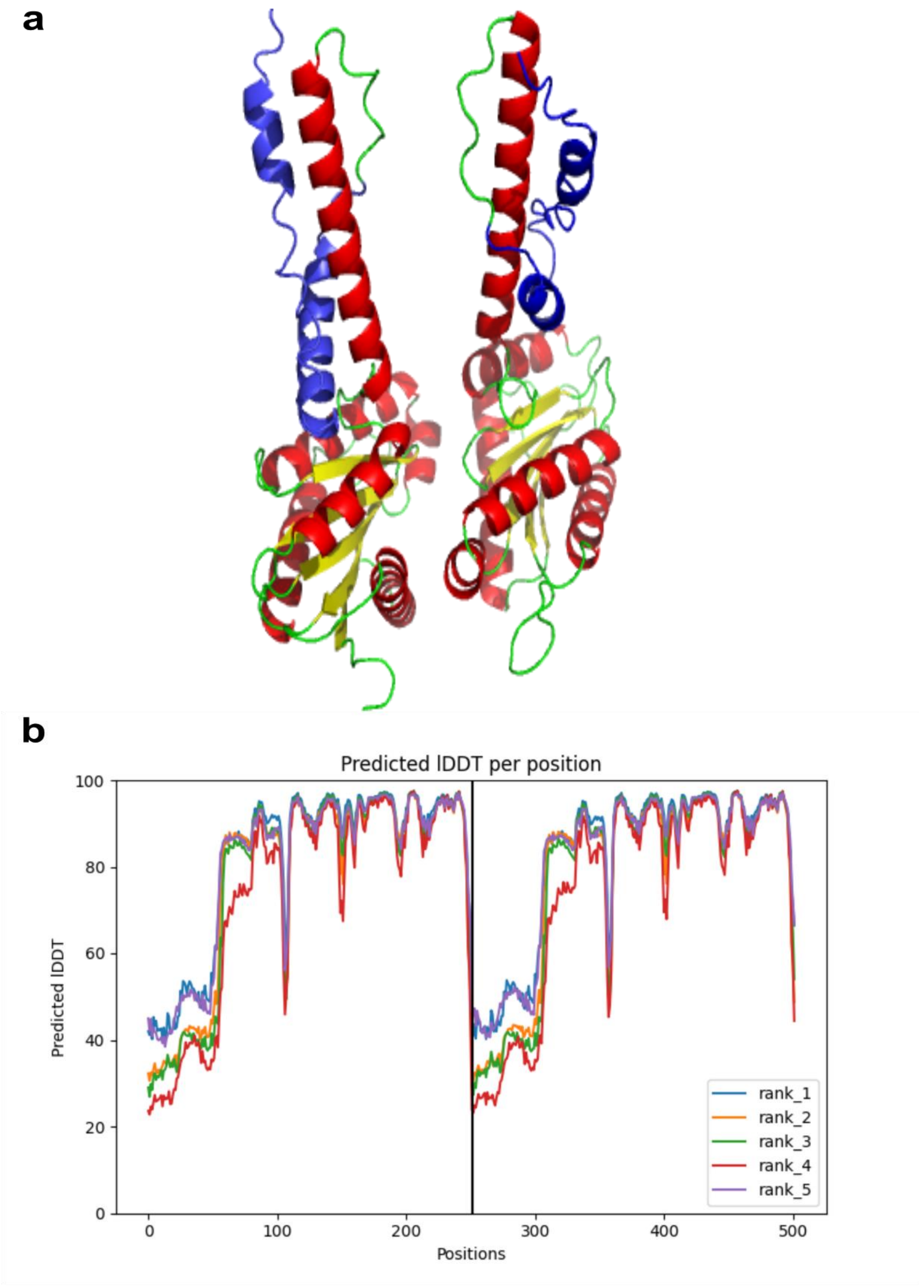

Figure S1. **a**. ColabFold structure prediction of BB3903 dimer. Alpha helixs in red and β sheetsin yellow. In blue the predicted N term disordered region. **b.** Predicted IDDT of the five predicted dimers.

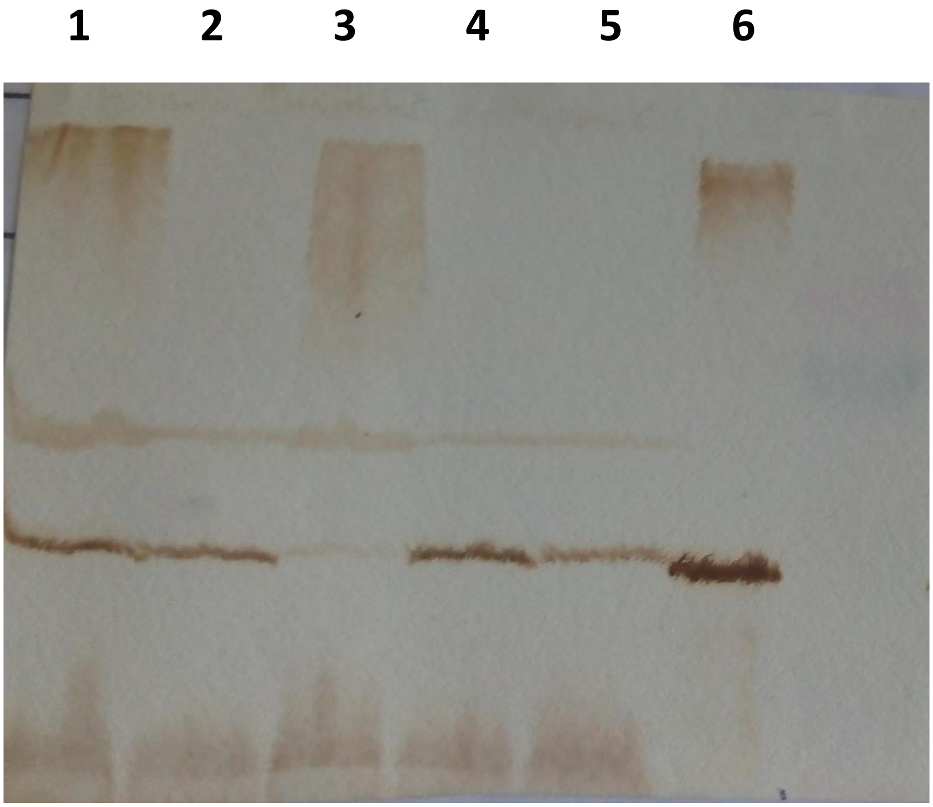

Figure S2. Non cropped western blot of *B. bronchiseptica* strains shown in Figure 2A. (1) *Bb*-pEmpty (2) *Bb*Δ*bdcB*-pEmpty, (3) *Bb*-p*bdcB*, (4) *Bb*-pΔN-*bdcB*, (5) *Bb*-p*bdcB*(GAAF), (6) purified flagellin

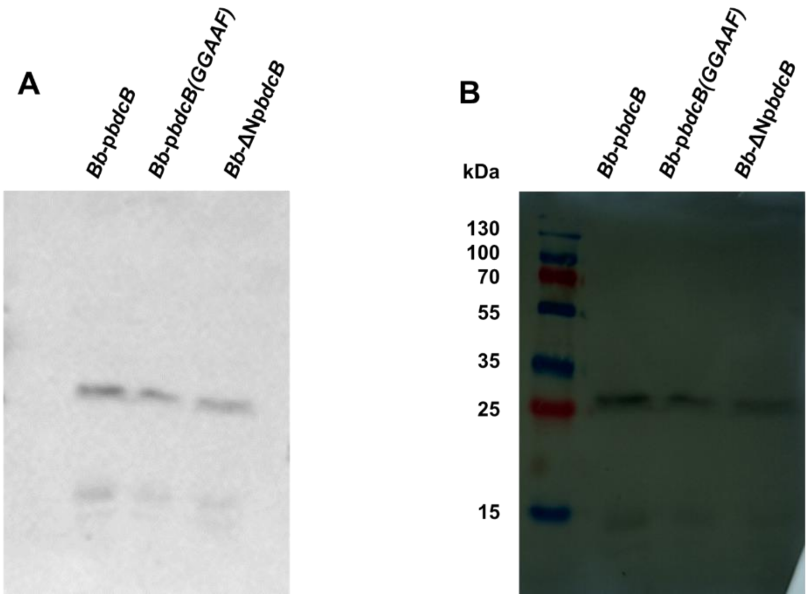

Figure S3. **A.** Western blot analysis of *B. bronchiseptica* expressing from a plasmid *bdcB*, *bdcB(GGAAF)*, or ΔN-*bdcB* with an HA tag. An expected band of approximately 28 kDa was observed in all lines (arrow). **B.** Superimpose of western blot and PVDF membrane. Marker from Thermo Scientific Page Ruler™ Plus.

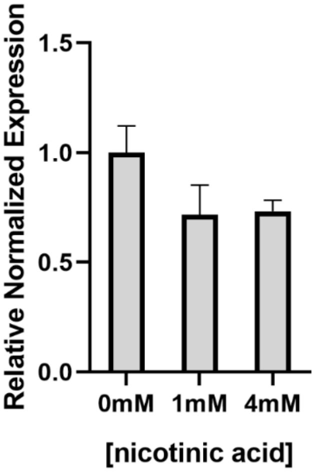

Figure S4. Relative normalized expression of *bdcB* in different nicotinic acid concentrations. RNA was extracted from ON SS culture with indicated nicotinic acid addition. qRT-PCR was performed, and results were analyzed by the ΔΔ method, as described in Materials and Methods. Results are average of three independent experiments.

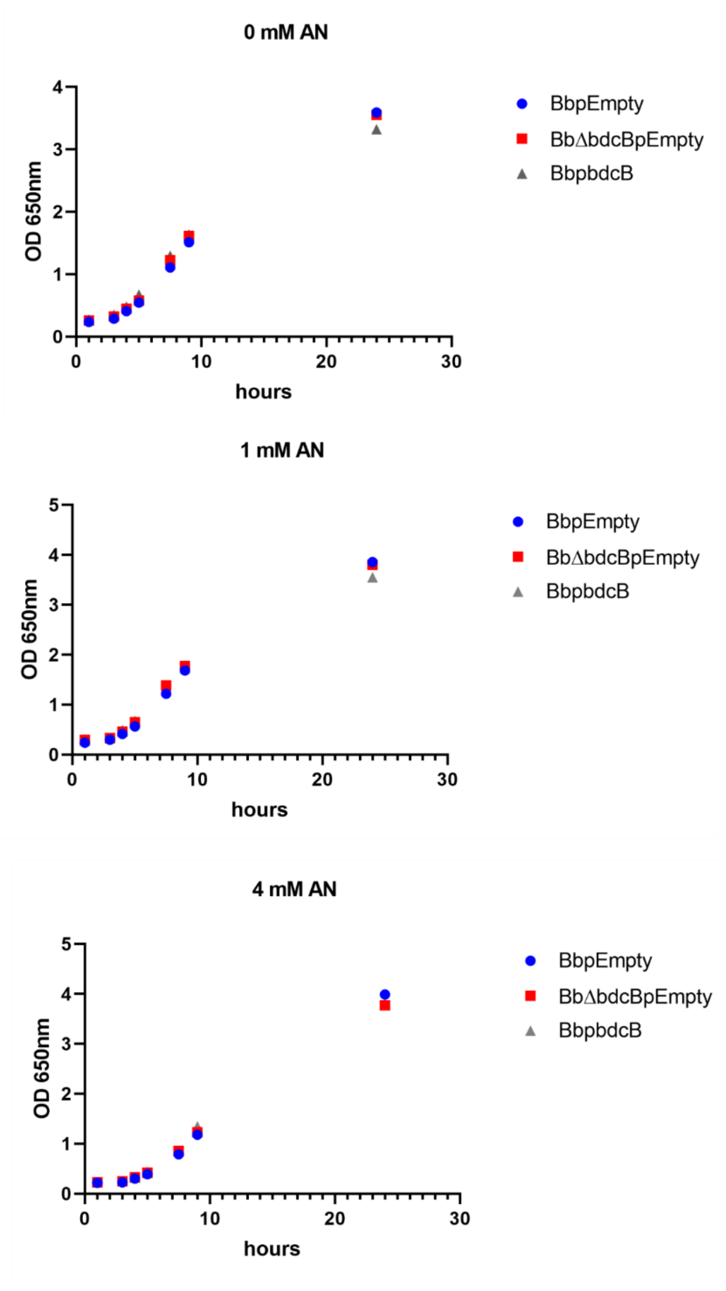

Figure S5. Growth of *B. bronchiseptica* strains in SS media. Flasks with SS media supplemented with nicotinic acid if necessary were inoculated with *B. bronchiseptica* strains at an initial DO_{650nm} = 0.1 and incubated at 37°C and 160 rpm. At the indicated time DO_{650nm} was measured. Results are representative of three experiments.

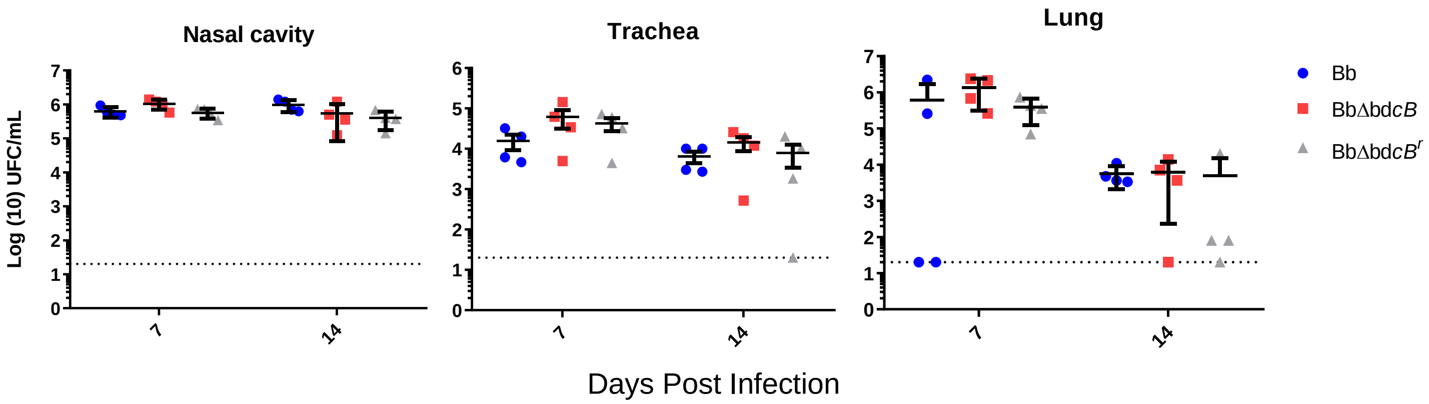

Figure S6. C57BL/6J mice were intranasally inoculated as described in Figure 3.

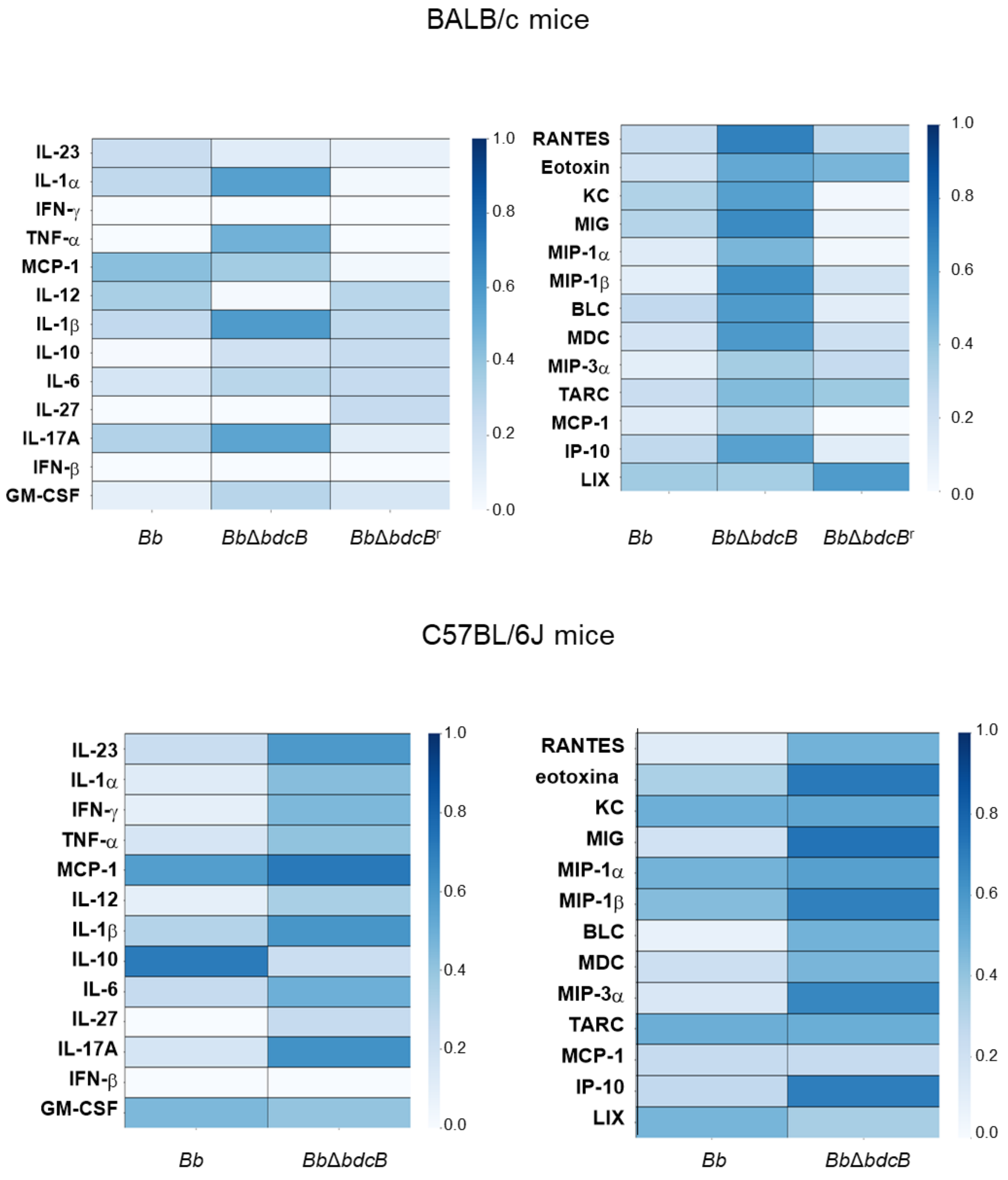

Figure S7. Heat map representation of cytokines concentration in lung homogenate supernatants from infected BALB/c and C57BL/6J mice, 7 days post-infection.

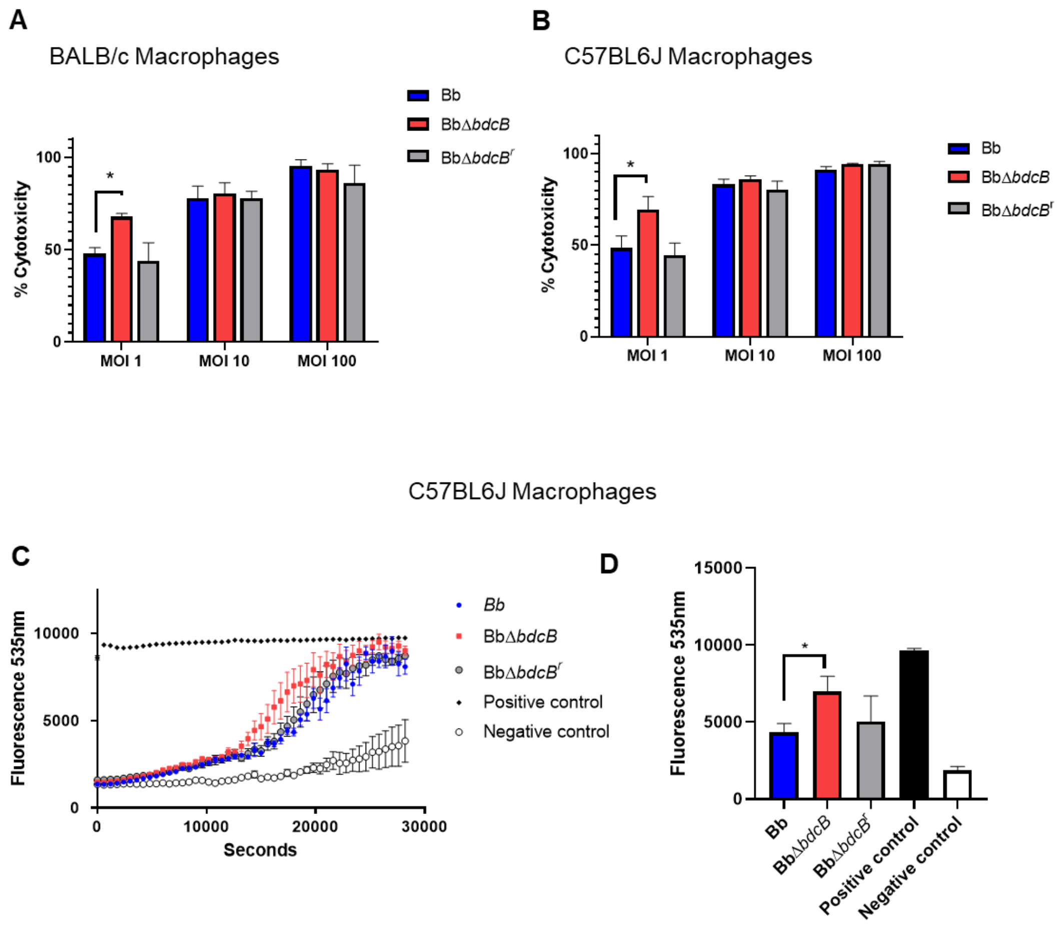

Figure S8. Cytotoxicity assays on BALB/c (**A**) or C57BL/6J (**B**) derived macrophages. *Bb*, *Bb*Δ*bdcB*, and *Bb*Δ*bdcB* strains were added at indicated MOI on macrophage monolayers and then incubated in 5% CO_2 at 37°C. Cytotoxicity assays at 4 hours were conducted using a Pierce LDH cytotoxicity assay kit (CytoTox 96^{R} non-radioactive cytotoxicity assay protocol, Promega), and results were expressed relative to the maximum LDH release control. Three biologically independent experiments were performed. *, $p<0.05$ (unpaired one-tailed Student's t-test). (**C**) Cytotoxicity assays on C57BL/6J derived macrophages. *Bb*, *Bb*Δ*bdcB*, and *Bb*Δ*bdcB* strains were added at MOI=1 on macrophage monolayers and then incubated in 5% CO_2 at 37°C. The assay was performed in PI-containing media, fluorescence was measured at different time points at 635nm (**C**), and statistical analysis was performed at 5 hs (18000 seconds) (**D**). Three biologically independent experiments were performed. *, $p<0.05$ (unpaired one-tailed Student's t-test).

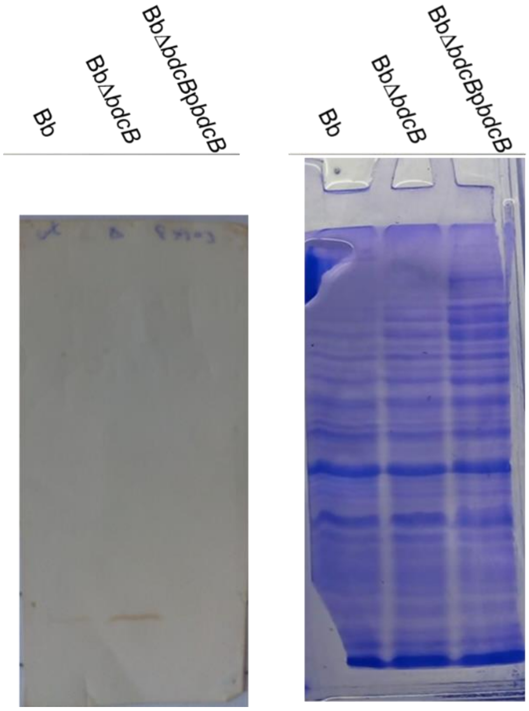

Figure S9. SDS PAGE gel stained with Coomassie blue solution and non cropped western blot shown in Figure 7B. Two biologically independent experiments were performed.

References

1. Robert M. Q. Shanks NCCSMHCMT and GAO. Saccharomyces cerevisiae-based molecular tool kit for manipulation of genes from gram-negative bacteria. *Appl Environ Microbiol* . 2006;72(7):5027-5036.

2. Newell PD, Yoshioka S, Hvorecny KL, Monds RD, O'Toole GA. Systematic analysis of diguanylate cyclases that promote biofilm formation by Pseudomonas fluorescens Pf0-1. *Journal of Bacteriology*. 2011;193(18):4685-4698. doi:10.1128/JB.05483-11

3. Sisti F, Ha DG, O'Toole GA, Hozbor D, Fernández J. Cyclic-di-GMP signaling regulates motility and biofilm formation in Bordetella bronchiseptica. *Microbiology (Reading)*. 2013;159(Pt 5):869-879. doi:10.1099/mic.0.064345-0

4. Karine Le Blay PGNGRC. Antigenic polymorphism of the lipopolysaccharides from human and animal isolates of Bordetella bronchiseptica. *Microbiology (Reading)* . 1997;143(4):1433-1441.

5. Tracy L Nicholson AMBETHSLB. Microarray and functional analysis of growth phase-dependent gene regulation in Bordetella bronchiseptica. *Infect Immun* . 2009;77(10):4221-4231.

6. Ahuja U, Shokeen B, Cheng N, et al. Differential regulation of type III secretion and virulence genes in Bordetella pertussis and Bordetella bronchiseptica by a secreted anti-σ factor. Published online 2016. doi:10.1073/pnas.1600320113

7. Ambrosis N, Fernández J, Sisti F. Counter-Selection Method for Markerless Allelic Exchange in Bordetella bronchiseptica Based on sacB Gene From Bacillus subtilis. Curr Protoc Microbiol. 2020 Dec;59(1):e125. doi: 10.1002/cpmc.125.
