# Supplementary figures and images for: Bordetella bronchiseptica diguanylate cyclase BdcB inhibits the type three secretion system and impacts the immune response
Source: Sci Rep. 2023 May 2;13:7157. doi: 10.1038/s41598-023-34106-x (PMC10154355; doi:10.1038/s41598-023-34106-x)

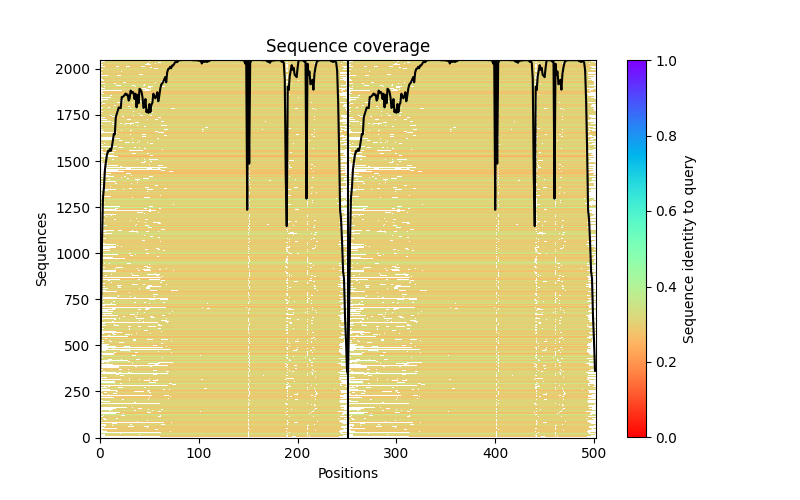

Supplement: Supplementary file 2 — Supplementary Information 2. [file 41598_2023_34106_MOESM2_ESM.zip › BB3903dimero_23b23_coverage.png]

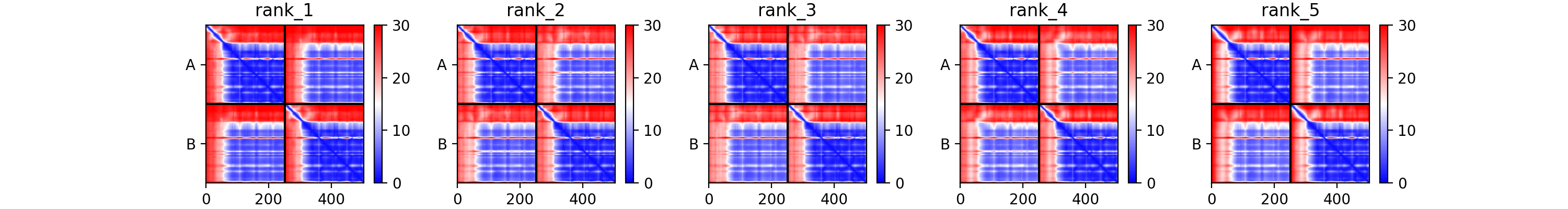

Supplement: Supplementary file 2 — Supplementary Information 2. [file 41598_2023_34106_MOESM2_ESM.zip › BB3903dimero_23b23_PAE.png]

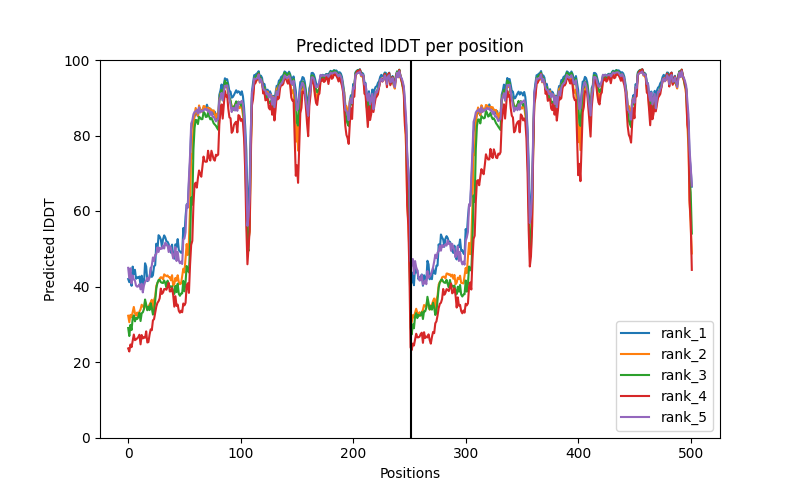

Supplement: Supplementary file 2 — Supplementary Information 2. [file 41598_2023_34106_MOESM2_ESM.zip › BB3903dimero_23b23_plddt.png]
